# WTAP-mediated m^6^A modification modulates bone marrow mesenchymal stem cells differentiation potential and osteoporosis

**DOI:** 10.1038/s41419-023-05565-x

**Published:** 2023-01-17

**Authors:** Yunhao You, Jincheng Liu, Lu Zhang, Xiang Li, Zhenqian Sun, Zihan Dai, Jinlong Ma, Guangjun Jiao, Yunzhen Chen

**Affiliations:** 1 Department of Orthopaedics, Qilu Hospital of Shandong University, 250012 Jinan, Shandong China; 2 The First Clinical College of Cheeloo College of Medicine, Shandong University, 250012 Jinan, Shandong China

**Keywords:** Osteoporosis, Stem-cell differentiation

## Abstract

An imbalance in the differentiation potential of bone marrow mesenchymal stem cells (BMSCs) is an important pathogenic mechanism underlying osteoporosis (OP). N6-methyladenosine (m^6^A) is the most common post-transcriptional modification in eukaryotic cells. The role of the Wilms’ tumor 1-associated protein (WTAP), a member of the m^6^A functional protein family, in regulating BMSCs differentiation remains unknown. We used patient-derived and mouse model-derived samples, qRT-PCR, western blot assays, ALP activity assay, ALP, and Alizarin Red staining to determine the changes in mRNA and protein levels of genes and proteins associated with BMSCs differentiation. Histological analysis and micro-CT were used to evaluate developmental changes in the bone. The results determined that WTAP promoted osteogenic differentiation and inhibited adipogenic differentiation of BMSCs. We used co-immunoprecipitation (co-IP), RNA immunoprecipitation (RIP), methylated RNA immunoprecipitation (MeRIP), RNA pulldown, and dual-luciferase assay to explore the direct mechanism. Mechanistically, the expression of WTAP increased during osteogenic differentiation and significantly promoted pri-miR-181a and pri-miR-181c methylation, which was recognized by YTHDC1, and increased the maturation to miR-181a and miR-181c. MiR-181a and miR-181c inhibited the mRNA expression of SFRP1, promoting the osteogenic differentiation of BMSCs. Our results demonstrated that the WTAP/YTHDC1/miR-181a and miR-181c/SFRP1 axis regulated the differentiation fate of BMSCs, suggesting that it might be a potential therapeutic target for osteoporosis.

## Introduction

Osteoporosis is a common systemic skeletal disorder characterized by low bone mass and destruction of the bone microstructure, gradually resulting in bone fragility and increased fracture risk [1–4]. During osteoporosis, the adipogenic differentiation of bone marrow mesenchymal stem cells (BMSCs) is enhanced, and osteogenic differentiation is weakened. An imbalance in the differentiation of BMSCs is the main cause of osteoporosis [5]. Understanding the mechanism of osteogenic differentiation of BMSCs holds the key to the development of therapeutic interventions for osteoporosis [6].

N6-methyladenosine (m^6^A) is the most common post-transcriptional RNA modification in eukaryotic cells and is carried out by the RNA methyltransferase complex, which comprises methyltransferase-like 3 (METTL3), methyltransferase-like 14 (METTL14), and Wilms’ tumor 1-associated protein (WTAP) [7–13]. This process can be reversed by “erasers,” such as demethylases, including alkylation repair homolog protein 5 (ALKBH5) and fat mass and obesity-associated protein (FTO) [14–18]. The transcripts modified with m^6^A could be identified by “readers,” such as YT521-B homology (YTH) domain-containing proteins (YTHDF1-3, YTHDC1, and YTHDC2) and insulin-like growth factor 2 mRNA-binding proteins (IGF2BPs; including IGF2BP1/2/3) to achieve the corresponding functional adjustment [19–23]. Several studies have demonstrated that m^6^A methylation regulates the balance between osteogenesis and adipogenesis of BMSCs [24–27], yet how m^6^A modification is involved in the pathogenesis of osteoporosis remains relatively unknown.

MicroRNAs (miRNAs) are small noncoding RNAs that negatively regulate gene expression by interacting with target mRNAs to destabilize them or restrain their translation [28–31]. MiRNAs regulate up to 60% of the human protein-coding genes. Several studies have reported that miRNAs are widely involved in bone proliferation, apoptosis, and inflammation [26, 27]. MiRNAs play a major role in regulating BMSCs differentiation, which seems to be closely correlated with the pathogenesis of osteoporosis [28]. MiR-26b regulates subchondral BMSCs through β-catenin and reduces the bone loss caused by abnormal occlusion [32]. Thus, further investigation of the function of miRNAs in the etiology, treatment, and prognosis of osteoporosis is urgently needed.

This study aimed to investigate whether the interaction between m^6^A modification and miRNAs could regulate the osteogenic differentiation ability of BMSCs and thus affect osteoporosis. Our study showed that the expression of WTAP was lower in patients with osteoporosis than in normal controls, and the expression of WTAP increased during osteogenic induction. Increasing the expression of WTAP significantly enhanced the m^6^A modification of pri-miR-181a and pri-miR-181c. After that, YTHDC1 recognizes the methylation modification of pri-miR-181a and pri-miR-181c and promotes its maturation. Elevated expression of miR-181a and miR-181c resulted in decreased expression of the target gene secreted frizzled-related protein 1 (SFRP1), which promoted the osteogenic differentiation of BMSCs. Our research revealed that WTAP could promote osteogenic differentiation and inhibit adipogenic differentiation of BMSCs through the YTHDC1/ miR-181a and miR-181c /SFRP1 axes, which could be of significance in developing therapeutic targets for osteoporosis.

## Results

### The expression of WTAP was decreased in the bone tissue of osteoporosis patients and ovariectomized mice

**Fig. 1:**
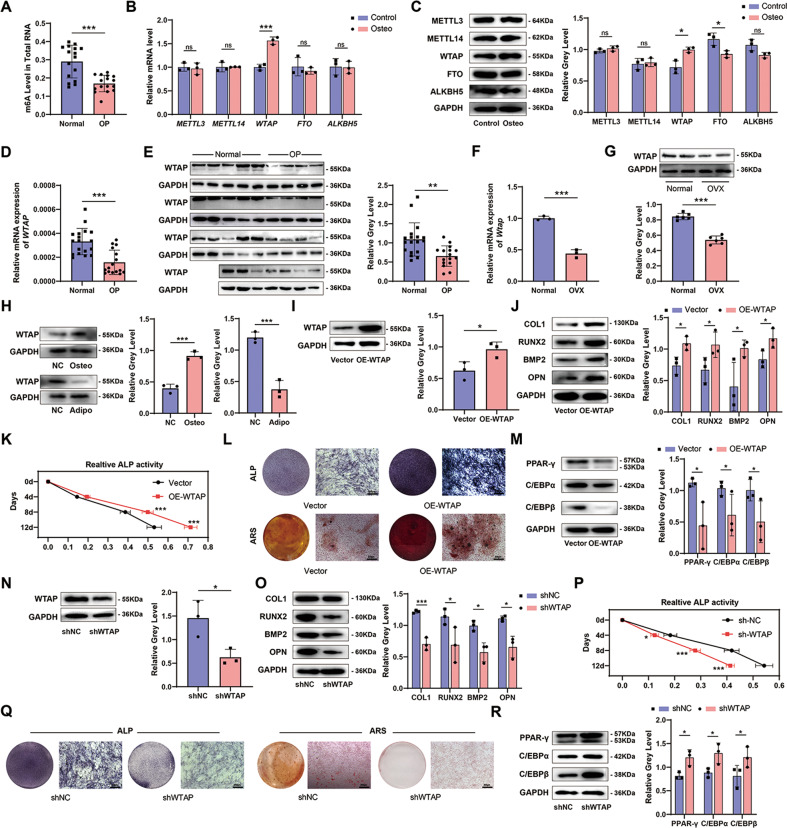
The expression of WTAP during osteoporosis and the function of WTAP in BMSCs differentiation. **A** Analysis of m^6^A level in total RNA in bone tissues of osteoporosis patients. **B**, **C** The mRNA and protein expression of methylase and demethylase of BMSCs on day 3 during osteogenic differentiation. **D**, **E** The mRNA and protein expression of WTAP in the bone tissue of normal and osteoporosis patients. **F**, **G** The mRNA and protein expression of WTAP in the bone tissue of normal and OVX mice. **H** The protein expression of WTAP on day 3 during osteogenic differentiation or adipogenic differentiation. **I** The protein expression of WTAP in the OE-WTAP primary mouse BMSCs and Vector BMSCs. **J** The protein expression of COL1, RUNX2, BMP2, and OPN in the OE-WTAP BMSCs and Vector BMSCs on day 3 during osteogenic differentiation. **K** Relative ALP activity in the OE-WTAP BMSCs and Vector BMSCs during osteogenic differentiation. **L** ALP staining on day 7 and ARS staining on day 14 in the OE-WTAP group and Vector group. **M** The protein expression of PPAR-γ, C/EBPα, and C/EBPβ on day 3 during adipogenic differentiation in the OE-WTAP group and Vector group. **N** The protein expression of WTAP in the shWTAP group and shNC group. **O** The protein expression of COL1, RUNX2, BMP2, and OPN in the shWTAP group and shNC group on day 3 during osteogenic differentiation. **P** Relative ALP activity in the shWTAP group and shNC group on a day during osteogenic differentiation. **Q** ALP staining on day 7 and ARS staining on day 14 in the shWTAP group and shNC group during osteogenic differentiation. **R** The protein expression of PPAR-γ, C/EBPα, and C/EBPβ on day 3 during adipogenic differentiation in the shWTAP group and shNC group. Data are expressed as the mean ± SD, *p < 0.05, **p < 0.01, ***p < 0.001.

To investigate the potential role of m^6^A methylation in osteogenic differentiation, we first compared m^6^A methylation levels in bone tissues of patients with osteoporosis and found that m^6^A levels were decreased compared to those in normal individuals (Fig. 1A). To elucidate the regulatory mechanism of m^6^A modification, we analyzed the expression of methylase and demethylase during osteogenic differentiation of BMSCs by qRT-PCR and WB, and the results showed that the expression of methylase *WTAP* was significantly increased (Fig. 1B, C). Therefore, we collected patient samples for verification, and both qRT-PCR and WB showed that the expression of WTAP in the bone tissues of osteoporosis patients was significantly decreased (Fig. 1D, E). We established an ovariectomized (OVX) mouse model of osteoporosis. The WTAP expression level in the bone tissue of OVX mice was also significantly reduced compared with that in the sham group after 2 months (Fig. 1F, G). These results indicate that WTAP expression is down-regulated in osteoporotic bone tissue. However, it is not clear how WTAP expression changes during BMSCs differentiation. Therefore, we used qRT-PCR and WB to determine whether WTAP was specifically up-regulated during osteogenic differentiation and down-regulated during adipogenic differentiation (Supplementary Fig. 1A and Fig. 1H).

### WTAP promoted osteogenic differentiation of BMSCs and impaired adipogenic differentiation

Next, we investigated the role of WTAP in BMSCs differentiation. The WTAP overexpression (OE-WTAP) virus was used to transfect mouse primary BMSCs, and the overexpression efficiency was confirmed by qRT-PCR and WB (Supplementary Fig. 1B and Fig. 1I). As shown in Supplementary Fig. 1C, OE-WTAP promoted the transcription of osteogenesis-related genes including collagen type I (*Col1*), runt-related transcription factor 2 (*Runx2*), alkaline phosphatase (*Alp*), bone morphogenetic protein-2 (*Bmp2*), and osteopontin (*Opn*) during osteogenic differentiation. The protein levels of COL1, RUNX2, BMP2, and OPN showed the same trend (Fig. 1J). Furthermore, ALP activity, ALP staining, and alizarin red staining showed that ALP activity and extracellular matrix mineralization were significantly increased in the OE-WTAP group (Fig. 1K, L). On the other hand, overexpression of WTAP decreased the adipogenic ability of BMSCs. Both mRNA (lipoprotein lipase(*Lpl*), adipocyte-specific fatty acid-binding protein (*aP2*), CCAAT/enhancer-binding protein α(*C/ebpα*), CCAAT/enhancer-binding protein-beta (*C/ebpβ*), peroxisome proliferator-activated receptor-γ (*Ppar-γ*) (Supplementary Fig. 1D)) and protein (PPAR-γ, C/EBPα, and C/EBPβ (Fig. 1M)) levels of adipogenic genes were decreased in OE-WTAP group during adipogenic differentiation. Lentivirus-mediated knockdown of WTAP (Supplementary Fig. 1E and Fig. 1N) reduced osteogenic gene expression (Supplementary Fig. 1F and Fig. 1O), ALP activity, and extracellular matrix calcification (Fig. 1P, Q) during osteogenic differentiation and increased the expression of adipogenic genes during adipogenic differentiation (Supplementary Fig. 1G and Fig. 1R). Our preliminary results suggest that WTAP can simultaneously increase the osteogenic potential of BMSCs and decrease their adipogenic potential.

### WTAP supplementation decreases bone loss induced by ovariectomy

**Fig. 2:**
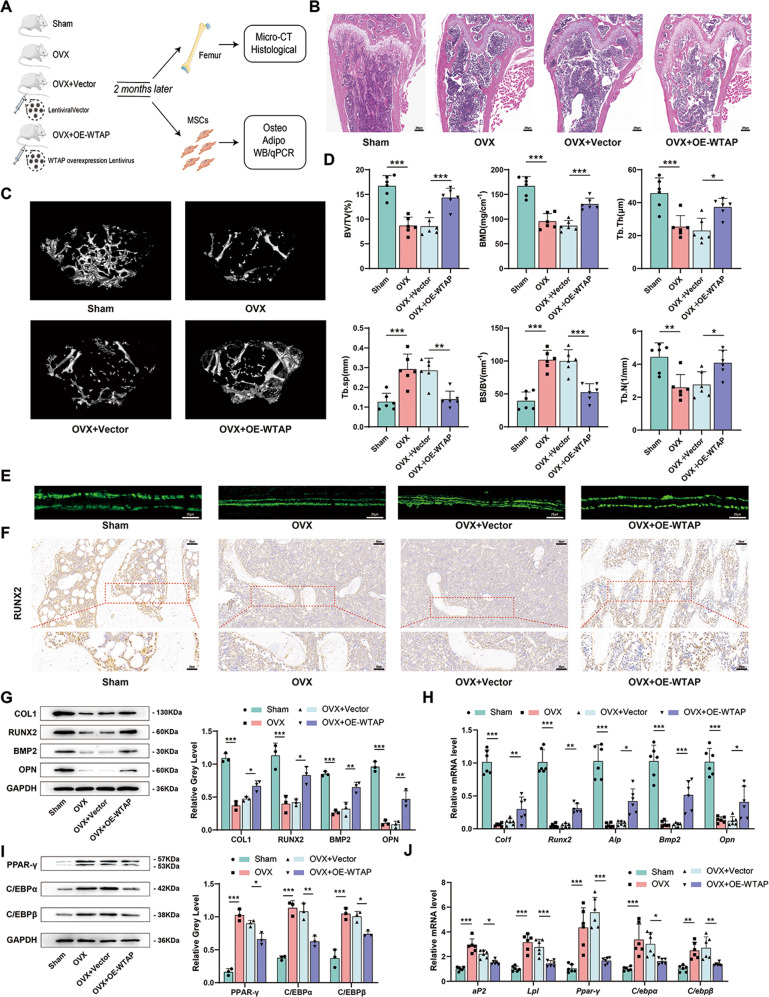
WTAP supplementation decreases bone loss induced by ovariectomy. **A** Scheme of the experimental strategy. In short, ovariectomized mice were treated with WTAP overexpression lentivirus or control lentivirus intravenously for 2 months. **B** Representative H&E staining of femurs in different mice group. **C** Representative micro-CT images of trabecular bone from the femoral metaphysis in different mice group. **D** bone volume per tissue volume (BV/TV, %), trabecular bone mineral density (BMD, g/cm^3^), trabecular thickness (Tb.Th, mm), trabecular separation (Tb.Sp, mm), Bone volume ratio (BS/BV) and trabecular number (Tb.N, 1/mm) analysis of the femurs of mice. **E** Calcein double-labeling analysis indicated osteoblastic bone formation. **F** Representative IHC images of Runx2 in the femur sections. **G** and **H** The protein or mRNA expression of osteogenic-related genes in primary BMSCs during osteogenic induction on day 3. **I** and **J** The protein or mRNA expression of adipogenic-related genes in primary BMSCs during adipogenic induction on day 3. Data are expressed as the mean ± SD, *p < 0.05, **p < 0.01, ***p < 0.001.

To evaluate the potential role of WTAP in bone loss induced by ovariectomy, ovariectomized 10-week-old mice were treated with an intravenous injection of WTAP overexpression lentivirus or control lentivirus starting 3 days after ovariectomy (Fig. 2A). WTAP expression decreased in the BMSCs of OVX mice and increased in OVX+ OE-WTAP mice 2 months later (Supplementary Fig. 2A). Bone mass was compared among sham-operated (Sham), ovariectomized (OVX), negative control lentivirus-treated OVX (OVX+ Vector), and WTAP-overexpressing lentivirus-treated OVX mice (OVX+ OE-WTAP). Hematoxylin-eosin (H&E) staining (Fig. 2B) and Masson’s staining (Supplementary Fig. 2B) revealed that trabecular bone was significantly less in the femurs of OVX mice than in those of Sham mice. Staining also showed that WTAP overexpression decreased trabecular bone loss in OVX+ Vector mice. Micro-CT analysis revealed that WTAP overexpression decreased ovariectomy-induced bone loss (Fig. 2C, D). In addition, advanced osteoblastic bone formation after WTAP overexpression was confirmed by calcein double-labeling analysis (Fig. 2E). WTAP overexpression increased COL1, RUNX2, BMP2, and OPN expression in femur samples from ovariectomized mice, as evidenced by immunohistochemical staining analysis (Fig. 2F and Supplementary Fig. 2C). To evaluate the effect of WTAP supplementation on the osteogenic and adipogenic differentiation of BMSCs, BMSCs were isolated from sham, OVX, OVX+ Vector, and OVX+ OE-WTAP mice, and their osteogenic and adipogenic differentiation activities were compared. WTAP overexpression not only advanced osteoblastic differentiation (Fig. 2G, H) but also dampened adipogenic differentiation (Fig. 2I, J) of BMSCs after ovariectomy. Taken together, these results demonstrate that WTAP supplementation decreases ovariectomy-induced bone loss.

### WTAP promoted osteogenic differentiation of BMSCs and impaired adipogenic differentiation of BMSCs by promoting the expression of miR-181a and miR-181c

**Fig. 3:**
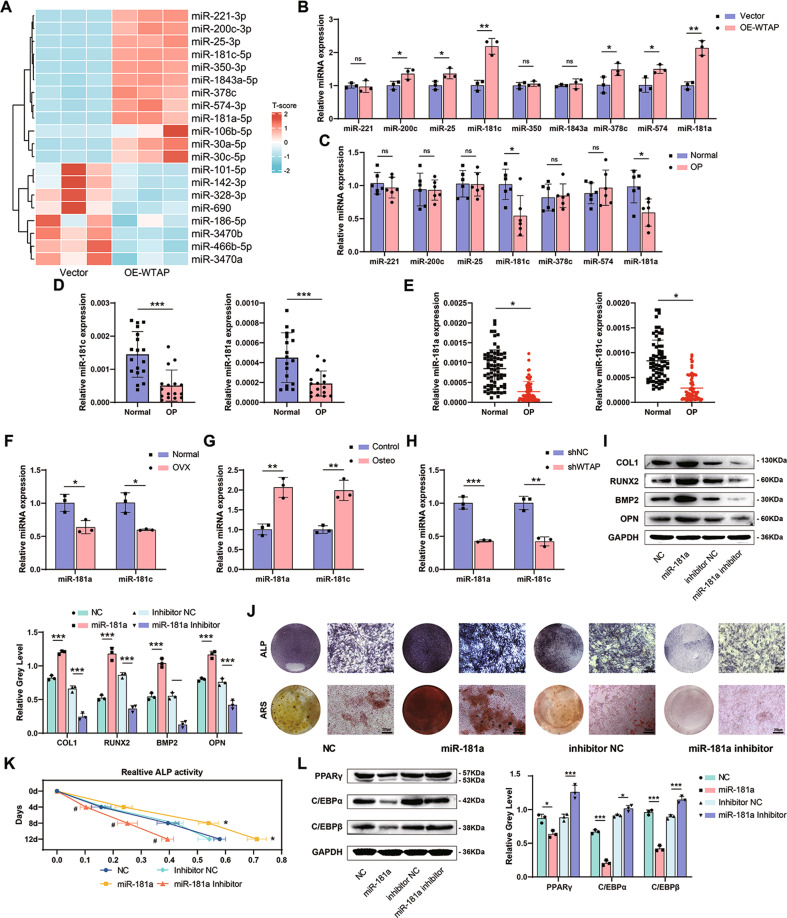
WTAP promoted osteogenic differentiation of BMSCs and impaired adipogenic differentiation of BMSCs by promoting the expression of miR-181a and miR-181c. **A** High-throughput miRNA sequencing of BMSCs in the Vector and OE-WTAP groups during osteogenic differentiation. **B** The relative expression of several miRNAs of BMSCs in the Vector and OE-WTAP groups during osteogenic differentiation determined by qRT-PCR. **C** The relative expression of several miRNAs of bone samples from osteoporosis patients determined by qRT-PCR. **D** The expression of miR-181a and miR-181c in the bone tissue of osteoporosis patients determined by qRT-PCR. **E** The expression of miR-181a and miR-181c in the circulating plasma of osteoporosis patients determined by qRT-PCR. **F** The expression of miR-181a and miR-181c in the femur of control mice and OVX mice was analyzed by qRT-PCR. **G** The expression of miR-181a and miR-181c of BMSCs on day 3 during osteogenic induction. **H** The expression of miR-181a and miR-181c of sh-NC or sh-WTAP BMSCs on day 3 during osteogenic induction. **I** The protein expression of osteogenic-related genes in primary BMSCs treated with NC, miR-181a, inhibitor NC, miR-181a inhibitor on day 3 during osteogenic induction. **J** and **K** ALP staining on day 7, ARS staining on day 14, and relative ALP activity of primary BMSCs treated with NC, miR-181a, inhibitor NC, miR-181a inhibitor during osteogenic induction. **L** The protein expression of adipogenic-related genes in primary BMSCs treated with NC, miR-181a, inhibitor NC, miR-181a inhibitor on day 3 during adipogenic induction. Data are expressed as the mean ± SD, *p < 0.05, **p < 0.01, ***p < 0.001.

It has been demonstrated that m^6^A methylation promotes post-transcriptional modification of miRNAs. To explore the mechanism by which WTAP regulates BMSCs differentiation, we performed high-throughput miRNA sequencing of BMSCs in the Vector and OE-WTAP groups during osteogenic differentiation (Fig. 3A). We used qRT-PCR to verify the sequencing results in BMSCs from mice in the Vector and OE-WTAP group and found 9 miRNAs were highly expressed after WTAP overexpression during osteogenic differentiation (Fig. 3B). We used bone samples from osteoporosis patients for verification and found that the expression levels of miR-181a and miR-181c were decreased in the bone tissues of the patients (Fig. 3C). Further evaluation showed that the expression of miR-181a and miR-181c in bone tissue (Fig. 3D) and circulating plasma (Fig. 3E) in osteoporosis patients was down-regulated. The same trend was observed in the femur bone of sham-operated (normal) and ovariectomized (OVX) mice (Fig. 3F). At the cellular level, we first conducted osteogenic induction of BMSCs and found that the expression of miR-181a and miR-181c increased (Fig. 3G). In contrast, the expression of miR-181a and miR-181c significantly decreased after WTAP knockdown (Fig. 3H).

Next, we explored the effects of miR-181a and miR-181c on the differentiation potential of BMSCs. BMSCs were treated with miRNA mimics or inhibitors, and gene interference efficiency was determined by qRT-PCR (Supplementary Fig. 3A). The results showed that miR-181a and miR-181c mimics increased the expression levels of osteogenic-related genes in BMSCS during osteogenic induction (Fig. 3I and Supplementary Fig. 3B, D, E). ALP activity and extracellular matrix mineralization were also significantly increased (Fig. 3J, K, and Supplementary Fig. 3F, G). In contrast, miR-181a and miR-181c inhibitors reduced the expression of osteogenic-related genes (Fig. 3I and Supplementary Fig. 3B, D, E), ALP activity, and extracellular matrix calcification (Fig. 3J, K, and Supplementary Fig. 3F, G) in BMSCs. During adipogenic induction, miR-181a and miR-181c mimics reduced the gene expression levels of adipogenic genes in BMSCs, while miRNA inhibitors increased those in BMSCs (Fig. 3L and Supplementary Fig. 3C, H, I).

### MiR-181a and miR-181c mimics and inhibitors rescued the effect of WTAP intervention on osteogenic differentiation of BMSCs

**Fig. 4:**
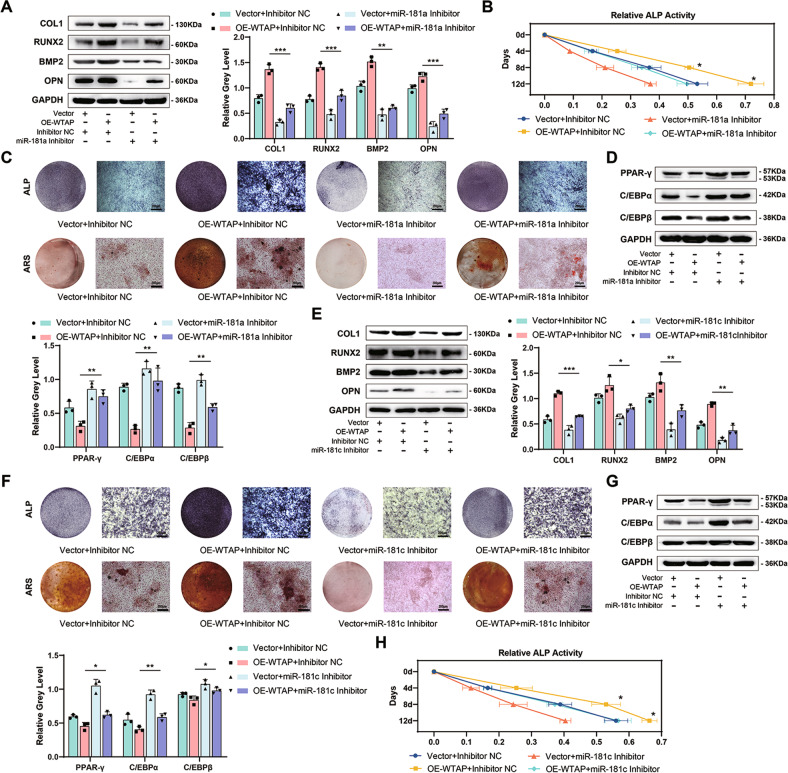
MiR-181a and miR-181c mimics and inhibitors rescued the effect of WTAP intervention on osteogenic differentiation of BMSCs. **A** The protein expression of osteogenic-related genes in primary BMSCs (Vector+inhibitor NC; OE-WTAP+inhibitor NC; Vector+miR-181a inhibitor; OE-WTAP+miR-181a inhibitor) on day 3 during osteogenic induction. **B** and **C** Relative ALP activity, ALP staining on day 7 and ARS staining on day 14 of primary BMSCs (Vector+inhibitor NC; OE-WTAP+inhibitor NC; Vector+miR-181a inhibitor; OE-WTAP+miR-181a inhibitor) during osteogenic induction. **D** The protein expression of adipogenic-related genes in primary BMSCs (Vector+inhibitor NC; OE-WTAP+inhibitor NC; Vector+miR-181a inhibitor; OE-WTAP+miR-181a inhibitor) on day 3 during adipogenic induction. **E** The mRNA or protein expression of osteogenic-related genes in primary BMSCs (Vector+inhibitor NC; OE-WTAP+inhibitor NC; Vector+miR-181c inhibitor; OE-WTAP+miR-181c inhibitor) on day 3 during osteogenic induction. **F** ALP staining on day 7 and ARS staining on day 14 of BMSCs (Vector+inhibitor NC; OE-WTAP+inhibitor NC; Vector+miR-181c inhibitor; OE-WTAP+miR-181c inhibitor) during osteogenic induction. **G** The mRNA or protein expression of adipogenic-related genes in primary BMSCs (Vector+inhibitor NC; OE-WTAP+inhibitor NC; Vector+miR-181c inhibitor; OE-WTAP+miR-181c inhibitor) on day 3 during adipogenic induction. **H** Relative ALP activity of primary BMSCs (Vector+inhibitor NC; OE-WTAP+inhibitor NC; Vector+miR-181c inhibitor; OE-WTAP+miR-181c inhibitor) during osteogenic induction. Data are expressed as the mean ± SD, *p < 0.05, **p < 0.01, ***p < 0.001.

When we transfected the WTAP-overexpressing cells with the miR-181a and miR-181c inhibitor, we observed a partial inhibition of the increased expression of osteogenic differentiation markers (Fig. 4A, E and Supplementary Fig. 4A, C) as well as increased ALP activity and extracellular matrix mineralization (Fig. 4B, C, F, H). The decreased expression of adipogenic differentiation-related markers was also partially rescued by miR-181a and miR-181c inhibitors (Fig. 4D, G, and Supplementary Fig. 4B, D).

When WTAP-knockdown cells were transfected with miR-181a and miR-181c mimics, we found that miR-181a and miR-181c mimics increased the expression of osteogenic differentiation markers (Supplementary Fig. 4E, F, K, L), ALP activity, and extracellular matrix mineralization (Supplementary Fig. 4G, H, M, N). However, adipogenic differentiation promoted by WTAP-knockdown cells was blocked by miR-181a and miR-181c mimics (Supplementary Fig. 4I, J, O, P).

In conclusion, WTAP affects the differentiation ability of BMSCS by altering the expression of miR-181a and miR-181c.

### WTAP-mediated m^6^A modification of pri-miR-181a and pri-miR-181c promotes its maturation

**Fig. 5:**
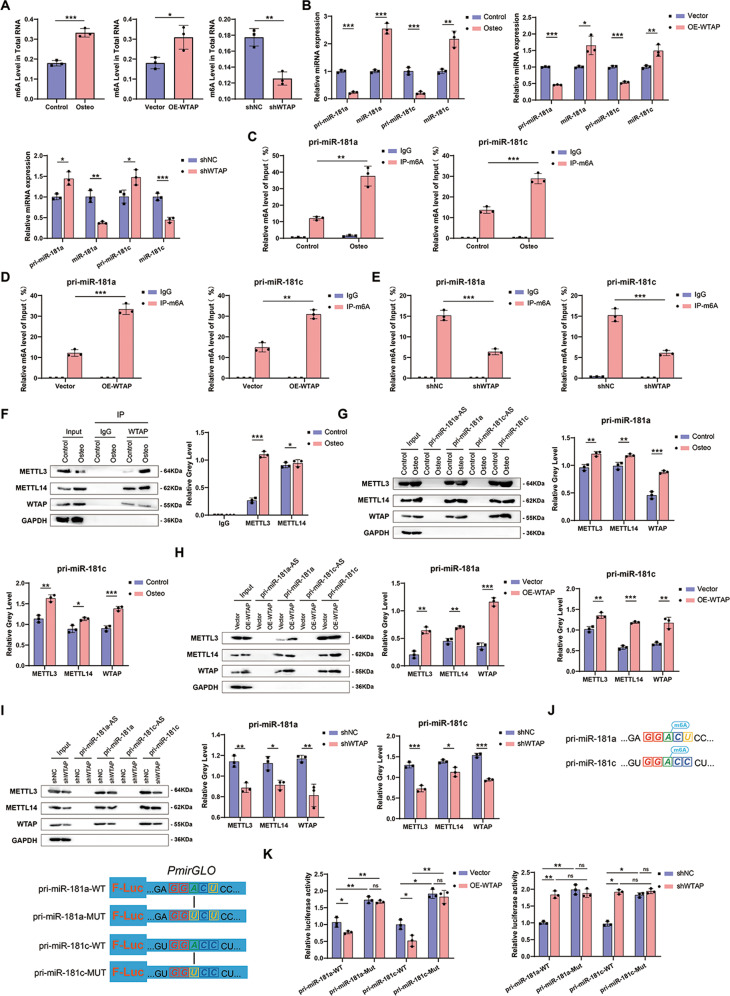
WTAP-mediated m^6^A modification of pri-miR-181a and pri-miR-181c promotes its maturation. **A** m^6^A level in total RNA of BMSCs during osteogenic differentiation, WTAP overexpression, and WTAP knockdown. **B** Relative expression of pri-miR-181a, pri-miR-181c, miR-181a, and miR-181c of BMSCs during osteogenic differentiation, WTAP overexpression, and WTAP knockdown. **C** m^6^A level of pri-miR-181a and pri-miR-181c in BMSCs during osteogenic differentiation by Me-RIP-qPCR. **D** m^6^A level of pri-miR-181a and pri-miR-181c in Vector or OE-WTAP BMSCs by Me-RIP-qPCR. **E** m^6^A level of pri-miR-181a and pri-miR-181c in sh-NC or sh-WTAP BMSCs by Me-RIP-qPCR. **F** Co-IP analysis of the interaction between METTL3, METTL14, and WTAP. **G** RNA pulldown of pri-miR-181a or pri-miR-181c with METTL3, METTL14, and WTAP in BMSCs during osteogenic differentiation, GAPDH was used as the loading control. **H** RNA pulldown of pri-miR-181a or pri-miR-181c with METTL3, METTL14, and WTAP in Vector or OE-WTAP BMSCs, GAPDH was used as the loading control. **I** RNA pulldown of pri-miR-181a or pri-miR-181c with METTL3, METTL14, and WTAP in sh-NC or sh-WTAP BMSCs, GAPDH was used as the loading control. **J** m^6^A sites in pri-miR-181a or pri-miR-181c and the corresponding mutant sites. **K** Dual-luciferase reporter assay of wild-type or site-mutant BMSCs with WTAP overexpression or WTAP knockdown. Data are expressed as the mean ± SD, *p < 0.05, **p < 0.01, ***p < 0.001, ns = not significant.

To explore whether WTAP regulates the expression of miR-181a and miR-181c through m^6^A modification, we measured m^6^A levels in cells and found that m^6^A levels increased during osteogenic differentiation, and the trend of m^6^A was in line with WTAP expression (Fig. 5A). Next, we studied the relationship between WTAP, miR-181a, and miR-181c expression. During osteogenic differentiation of BMSCs, pri-miR-181a and pri-miR-181c decreased, while miR-181a and miR-181c increased. As expected, when WTAP was overexpressed, pri-miR-181a and pri-miR-181c levels decreased, while miR-181a and miR-181c accumulated. However, when WTAP was knocked down, pri-miR-181a and pri-miR-181c accumulated, whereas mature miR-181a and miR-181c decreased (Fig. 5B). The results of MeRIP showed that osteogenic differentiation of MSCs led to the hypermethylation of pri-miR-181a and pri-miR-181c (Fig. 5C). Furthermore, MeRIP also showed that WTAP overexpression significantly increased the methylation of pri-miR-181a and pri-miR-181c (Fig. 5D), whereas WTAP knockdown significantly abrogated the methylation of pri-miR-181a and pri-miR-181c (Fig. 5E). These findings suggest that WTAP promotes the transition of pri-miR-181a and pri-miR-181c to mature miR-181a and miR-181c. The results of MeRIP also showed there are no m^6^A modifications on the miR-181a and miR-181c (Supplementary Fig. 5A–C). To elucidate the regulatory mechanism of m^6^A modification, we analyzed the expression of methylase and demethylase during osteogenic differentiation of BMSCs using WB, and the results showed that the expression of methylase WTAP was significantly increased (Fig. 1C). Co-IP analysis showed that METTL3 and METTL14 interacted with WTAP to form a methyltransferase complex during osteogenic differentiation (Fig. 5F). This may induce hypermethylation of pri-miR-181a and pri-miR-181c. Subsequently, we performed RNA pull-down and WB and detected osteogenic differentiation (Fig. 5G) and increased WTAP expression (Fig. 5H), promoting the interaction between pri-miR-181a and pri-miR-181c and methyltransferase complexes, including METTL3 and METTL14. Moreover, the knockdown of WTAP abolished the interaction of pri-miR-181a and pri-miR-181c with METTL3 and METTL14 (Fig. 5I). These results suggest that WTAP-mediated m^6^A modification of pri-miR-181a and pri-miR-181c contributes to the osteogenic differentiation of BMSCs.

Bioinformatics analysis revealed the presence of possible methylation sites GGACU and GGACC motifs in the exon region of pri-miR-181a and pri-miR-181c transcripts, respectively. A double luciferase reporter containing wild-type or mutant pri-miR-181a and pri-miR-181c sequences before firefly luciferase was constructed, in which A at the m^6^A site was replaced by U (Fig. 5J). The results showed that WTAP overexpression significantly reduced the luciferase activities of pri-miR-181a and pri-miR-181c wild-type reporters compared to mutant-type reporters. Knockdown of WTAP increased the luciferase activities of the pri-miR-181a and pri-miR-181c wild-type reporters (Fig. 5K). Luciferase activity showed that the point mutation decreased the modification of pri-miR-181a and pri-miR-181c by WTAP. These results suggest that increased m^6^A modification by WTAP up-regulates pri-miR-181a and pri-miR-181c maturation.

### YTHDC1-mediated methylation recognition promoted the maturation of pri-miR-181a and pri-miR-181c

**Fig. 6:**
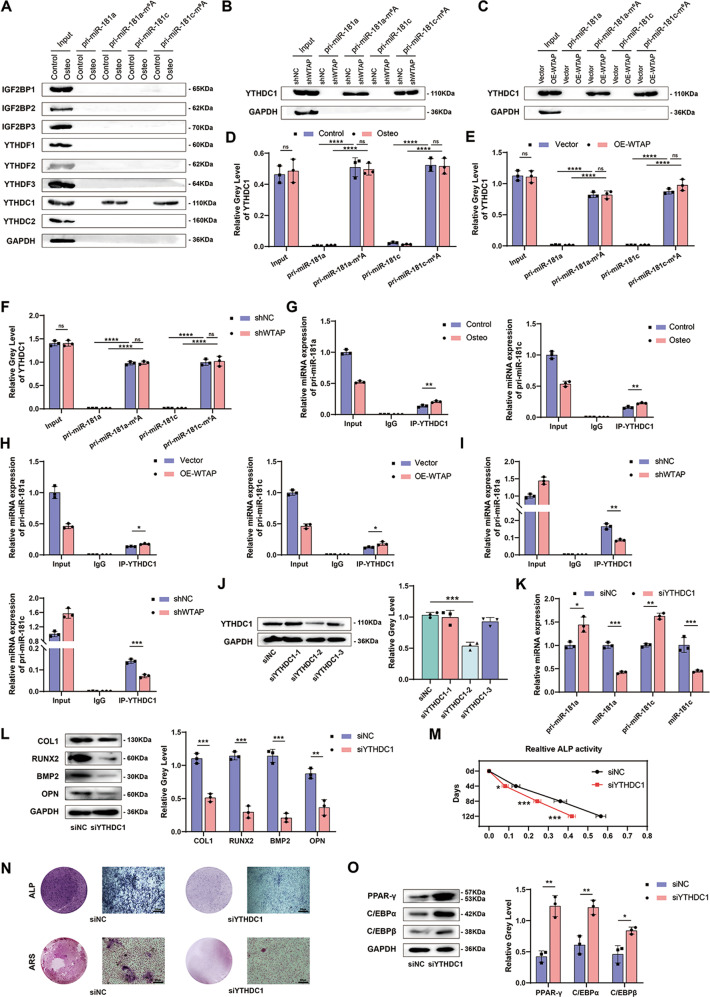
YTHDC1-mediated methylation recognition promoted maturation of pri-miR-181a and pri-miR-181c. **A** RNA pull-down of pri-miR-181a or pri-miR-181c with m^6^A readers in BMSCs during osteogenic differentiation, GAPDH was used as the loading control. **B** and **C** RNA pull-down of pri-miR-181a or pri-miR-181c with YTHDC1 in BMSCs with WTAP overexpression or WTAP knockdown, GAPDH was used as the loading control. **D**–**F** Analysis of RNA pull-down assay in BMSCs with osteogenic induction, WTAP overexpression, and WTAP knockdown. **G**–**I** RIP-qPCR analysis of pri-miR-181a or pri-miR-181c with YTHDC1 in BMSCs during osteogenic differentiation, WTAP overexpression, and WTAP knockdown. **J** The protein level of YTHDC1 of BMSCs with siYTHDC1 or siNC. **K** The relative expression of pri-miR-181a, pri-miR-181c, miR-181a, and miR-181c in BMSCs with siYTHDC1 or siNC analyzed by qRT-PCR. **L** The protein expression of osteogenic-related genes in primary BMSCs with siNC or siYTHDC1 on day 3 during osteogenic induction. **M** and **N** Relative ALP activity, ALP staining on day 7, and ARS staining on day 14 of primary BMSCs with siNC or siYTHDC1 during osteogenic induction. **O** The protein expression of adipogenic-related genes in primary BMSCs with siNC or siYTHDC1 on day 3 during adipogenic induction. Data are expressed as the mean ± SD, *p < 0.05, **p < 0.01, ***p < 0.001, ns = not significant.

To elucidate the regulatory mechanism of WTAP on pri-miR-181a and pri-miR-181c, we investigated whether readers are involved in regulating miR-181a and miR-181c biogenesis. RNA pull-down and WB showed that the expression of the readers did not change significantly during osteogenic induction. WB also showed that pri-miR-181a-m^6^A and pri-miR-181c-m^6^A instead of pri-miR-181a and pri-miR-181c were directly bound to YTHDC1 (Fig. 6A, D and Supplementary Fig. 5D). However, osteogenic differentiation did not increase the expression of YTHDC1 or the interaction between pri-miR-181a-m^6^A, pri-miR-181c-m^6^A, and YTHDC1 (Fig. 6A, D). Similar results were found in WTAP overexpression cells (Fig. 6B, E) and WTAP-knockdown cells (Fig. 6C, F). In addition, RIP confirmed the direct coupling of YTHDC1 with pri-miR-181a and pri-miR-181c (Fig. 6G). Moreover, the interaction increased in WTAP overexpression cells (Fig. 6H) and decreased in WTAP-knockout cells (Fig. 6I). Therefore, we hypothesized that YTHDC1 was a ‘reader’ of pri-miR-181a and pri-miR-181c after methylation, although its expression did not change with osteogenic differentiation.

To confirm this hypothesis, we conducted functional tests. First, we screened three small interference RNAs for knocking out YTHDC1 and chose siYTHDC1-2, with the highest knockout efficiency, for all subsequent experiments (Fig. 6J and Supplementary Fig. 5E). We found that the expression of pri-miR-181a and pri-miR-181c in the cells was increased, and the expression of miR-181a and miR-181c was decreased with YTHDC1 knockout (Fig. 6K). We also conducted functional experiments and concluded that the osteogenic differentiation ability of MSCS after YTHDC1 knockout was weakened (Fig. 6L–N and Supplementary Fig. 5F), and the adipogenic differentiation potential was enhanced (Fig. 6O and Supplementary Fig. 5G), consistent with the expression of miR-181a and miR-181c.

We then conducted phenotypic recovery assays to determine whether WTAP promotes miR-181a and miR-181c expression via YTHDC1. Knockdown of YTHDC1 in BMSCs restored the changes induced by the overexpression of WTAP, including miR-181a and miR-181c expression (Supplementary Fig. 5H), osteogenic differentiation potential (Supplementary Fig. 5I-L) as well as adipogenic differentiation potential (Supplementary Fig. 5M, N). This suggests that WTAP regulates miR-181a and miR-181c expression through YTHDC1. On the other hand, transfection with miR-181a (Supplementary Fig. 6A–F) and miR-181c mimics (Supplementary Fig. 6G–L) restored the effect of YTHDC1 knockout on the differentiation ability of BMSCs, suggesting that YTHDC1 knockdown regulated the differentiation of BMSCs through miR-181a and miR-181c. Therefore, our data proved that YTHDC1 is an indispensable regulator of WTAP that promotes pri-miR-181a and pri-miR-181c biogenesis.

### SFRP1 is a common downstream target of miR-181a and miR-181c in BMSCs

**Fig. 7:**
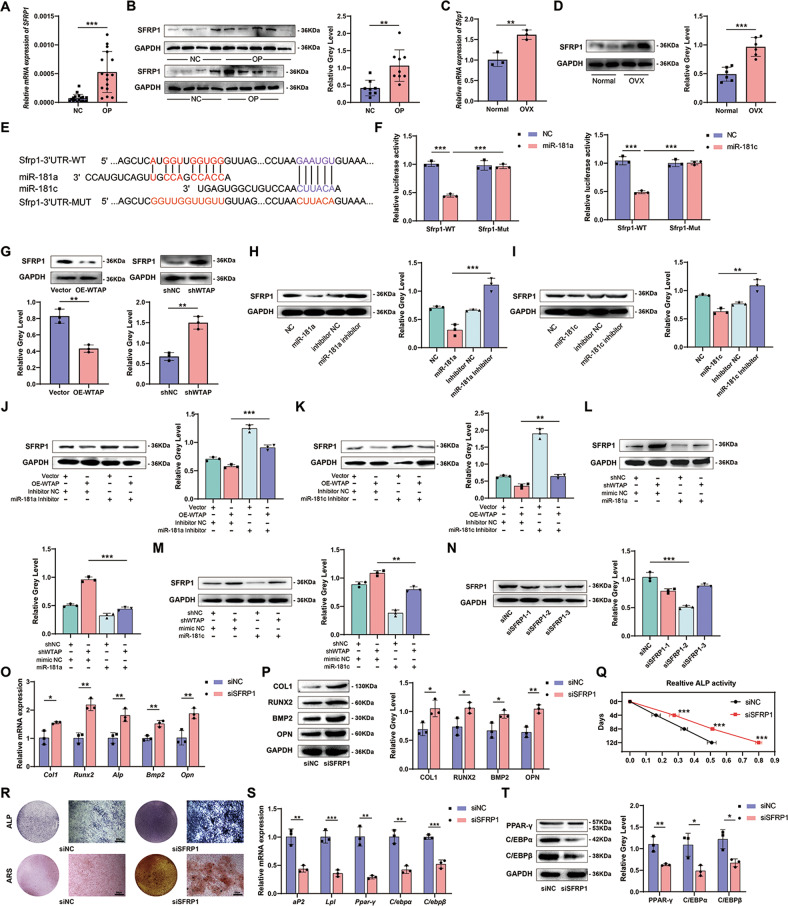
SFRP1 is a common downstream target of miR-181a and miR-181c in BMSCs. **A** and **B** The mRNA or protein expression of SFRP1 in bone tissues of normal or osteoporosis patients. **C** and **D** The mRNA or protein expression of SFRP1 in bone tissues of ovariectomized mice or control mice. **E** The binding sites of miR-181a and miR-181c in the 3’UTR of SFRP1 mRNA and the wide type or mutant type of binding site were shown. **F** Relative luciferase activity of the effect of miR-mimics on the SFRP1-WT and SFRP1-Mut reporters in BMSCs was measured by the dual luciferase reporter gene. **G**–**I** The protein expression of SFRP1 in BMSCs with WTAP, miR-181a, or miR-181c manipulation. **J**–**M** The protein expression of SFRP1 in BMSCs with WTAP and miR manipulation simultaneously. **N** The protein level of SFRP1 of BMSCs with siSFRP1 or siNC. **O** and **P** The mRNA or protein expression of osteogenic-related genes in primary BMSCs with siNC or siSFRP1 on day 3 during osteogenic induction. **Q** and **R** Relative ALP activity, ALP staining on day 7, and ARS staining on day 14 of primary BMSCs with siNC or siSFRP1. **S** and **T** The mRNA or protein expression of adipogenic-related genes in primary BMSCs with siNC or siSFRP1 on day 3 during adipogenic induction. Data are expressed as the mean ± SD, *p < 0.05, **p < 0.01, ***p < 0.001.

miRNAs inhibit gene expression mainly by binding and silencing the 3′-UTR region. Therefore, we used TargetScan and mirWalk to screen for possible downstream targets of miR-181a and miR-181c, respectively. We focused on SFRP1 because of its important role in the osteogenic differentiation of BMSCs. The expression of SFRP1 in osteoporotic bone tissues was higher than in normal tissue samples (Fig. 7A, B). Similar results were observed in the bone tissues of ovariectomized mice (Fig. 7C, D). To further explore the direct relationship between miR-181a, miR-181c, and SFRP1, we constructed a dual-luciferase reporter gene containing the 3′UTR of SFRP1 and the mutant 3′UTR of SFRP1 (Fig. 7E). The results showed that miR-181a and miR-181c mimics significantly reduced the luciferase activity of the SFRP1 wild-type reporter gene compared to that of the mutant reporter gene (Fig. 7F). Manipulating the expression of WTAP, miR-181a, and miR-181c showed that the expression of SFRP1 was directly related to phenotypic changes (Fig. 7G–I and Supplementary Fig. 7A–D). We also manipulated the expression of WTAP and miR simultaneously, and the results showed that the decreased expression of SFRP1 in the OE-WTAP group could be reversed at the mRNA and protein levels in cells co-transfected with OE-WTAP and miR inhibitors. In contrast, the increase in SFRP1 levels in the shWTAP group was reversed in cells co-transfected with shWTAP and miR mimics (Fig. 7J–M and Supplementary Fig. 7E–H). We then directly knocked down SFRP1 expression (Fig. 7N and Supplementary Fig. 7I), which resulted in a decrease in the osteogenic differentiation potential (Fig. 7O–R) and an increase in the adipogenic differentiation ability (Fig. 7S, T) of BMSCs. We simultaneously transfected siSFRP1, miR-181a, and miR-181c inhibitors into cells and found that phenotypic changes caused by miR-181a and miR-181c inhibitors were restored by siSFRP1 (Supplementary Fig. 7J–U). These results indicate that miR-181a and miR-181c can directly bind to the 3’UTR of SFRP1, promote osteogenic differentiation, and inhibit adipogenic differentiation by negatively regulating the expression of SFRP1. These results demonstrated that the WTAP/YTHDC1/miR-181a and miR-181c/SFRP1 axes promote osteogenic differentiation and inhibit the adipogenic differentiation of BMSCs.

## Discussion

Bone is continuously remodeled in a dynamic process in which osteoblasts are responsible for bone formation [33]. Osteoblasts are mainly derived from BMSCs in the bone marrow stroma and have the potential to differentiate into various functional cell types under certain conditions. Promoting the osteogenic differentiation of BMSCs is crucial for treating metabolic bone diseases like osteoporosis [2].

m^6^A regulates various pathological and physiological processes, including the multidirectional differentiation of BMSCs [24]. Many studies have shown that m^6^A modification regulates the balance between the osteogenic and adipogenic differentiation of BMSCs [15, 26, 27, 31, 32, 34]. For example, Mettl3 overexpression in BMSCs protected mice from estrogen deficiency-induced osteoporosis [26]. The m^6^A “reader,” YTHDF1, can promote the osteogenesis of BMSCs through translational regulation of ZNF839 [22]. Alkbh1-mediated DNA N6-methyladenine modification promotes osteogenic differentiation of BMSCs [18]. FTO regulates adipogenesis by controlling the cell cycle via an m^6^A-YTHDF2-dependent mechanism [16].

However, the mechanism by which WTAP regulates BMSCs differentiation of BMSCs is not well-studied [35–37]. WTAP is an important member of the m^6^A methyltransferase complex of cellular mRNA [35–40]. Most mRNAs bound to WTAP and METTL3 contain a consensus m^6^A motif [9, 10]. The RNA-binding capacity of METTL3 was greatly reduced in the absence of WTAP, suggesting that WTAP may play a role in regulating the recruitment of the m^6^A methyltransferase complex to mRNA targets [41].

We found that WTAP was up-regulated during osteogenic differentiation and down-regulated during the adipogenic differentiation of BMSCs. In addition, WTAP was significantly down-regulated in the bone tissue of patients with osteoporosis. Functional studies revealed that WTAP promoted osteogenic differentiation and inhibited adipogenic differentiation of BMSCs both in vitro and in vivo.

To explore the mechanism by which WTAP regulates the osteogenic differentiation of BMSCs, we conducted miRNA sequencing and found that miR-181a and miR-181c were highly expressed when WTAP was overexpressed. Some studies have confirmed the role of miR-181c in osteoporosis diagnosis [42, 43], but the mechanism underlying the changes in expression has not yet been investigated. Further, the correlation between miR-181a and miR-181c and BMSCs differentiation has not been clarified. We measured the expression of miRNAs in the plasma and bone tissue and found that miR-181a and miR-181c could be used as diagnostic markers of osteoporosis. miR-181a and miR-181c promoted osteogenic differentiation and inhibited adipogenic differentiation of BMSCs.

Furthermore, we found that WTAP was significantly up-regulated and interacted with METTL3 and METTL14 to increase the formation of the methyltransferase complex during osteogenic differentiation, thereby enhancing m^6^A modification of pri-miR-181a and pri-miR-181c. The methylation sites of pri-miR-181a and pri-miR-181c were confirmed by a luciferase assay, which also indicated that WTAP promoted the expression of miR-181a and miR-181c by methylating pri-miR-181a and pri-miR-181c.

mRNA transcripts with m^6^A modifications are typically regulated by YTH or IGF2BP [19–23, 44]. To elucidate the m^6^A modified reader, we performed an RNA pull-down assay and confirmed that YTHDC1 is a reader of pri-miR-181a and pri-miR-181c. We demonstrated that YTHDC1 depletion resulted in an overall reduction of miR-181a and miR-181c and concomitant accumulation of pri-miR-181a and pri-miR-181c in BMSCs, suggesting that YTHDC1-mediated decay of pri-miR-181a and pri-miR-181c mRNA is, at least in part, due to the reduced stability of its mRNA transcript.

MiRNAs generally inhibit the expression of target genes by binding to the 3′-UTR region [29]. We identified SFRP1 as a downstream target of miR-181a and miR-181c. SFRP1 is an antagonist of the Wnt signaling pathway, which is important for maintaining bone homeostasis. SFRP1 deficiency results in reduced apoptosis of osteoblasts and osteocytes [42, 45]. Therefore, WTAP-mediated up-regulation of miR-181a and miR-181c further regulated BMSCs differentiation by negatively regulating the expression of SFRP1.

However, this study has many limitations. For example, the mechanism of WTAP up-regulation during osteogenic differentiation has not yet been fully elucidated. WTAP itself, as a protein-coding gene, can be modified by methylation, acetylation, or other post-transcriptional regulators. Although METTL3 and METTL14 were not up-regulated during osteogenic differentiation, their binding to WTAP was enhanced. It is unclear how METTL3 and METTL14 are involved in BMSCs differentiation together with WTAP. Further studies are needed to elucidate the important role of m^6^A modification in BMSCs differentiation.

In summary, our study proposed a mechanism of WTAP in the process of BMSCs differentiation and revealed that WTAP up-regulated the m^6^A modification of pri-miR-181a and pri-miR-181c. Furthermore, the expression levels of miR-181a and miR-181c were increased by YTHDC1, which resulted in the downregulation of SFRP1 expression and osteogenic differentiation. These results suggest that WTAP may serve as a potential therapeutic target for metabolic bone disease.

## Methods

### Human specimens

The specimens were obtained from female patients and control subjects without osteoporosis or other bone-related anomalies. All subjects with other diseases and smoking or drinking histories were excluded. All participants signed written informed consent. Specimen collection was conducted by the Department of Orthopedics, Qilu Hospital of Shandong University. This study was approved by the Medical Ethics Committee of the Qilu Hospital of Shandong University.

### Ethics statement

We collected serum and bone samples from female OP patients and healthy female volunteers without OP and other bone-related anomalies from the Department of Orthopedics, Qilu Hospital of Shandong University (Jinan, Shandong, China). The experiments were approved by the Medical Ethics Committee of Shandong University Qilu Hospital.

### Cell culture

All mice used in this study were maintained in a specific pathogen-free (SPF) facility. After anesthesia, primary BMSCs were isolated from the tibia and femur of 8-week-old mice by flushing the bone marrow with α-minimum essential medium (α-MEM). Cells were seeded in 100 mm culture dishes and cultured in α-MEM. After 16 h of culture, the adherent cells were harvested for BMSCs culture. Osteogenesis was induced using 10 mM β-glycerophosphate, 10 nM dexamethasone, and 50 μg/mL ascorbic acid. Adipogenesis was induced using 10 mg/mL insulin, 500 mmol/L methyl isobutyl xanthine, and 1 μmol/L dexamethasone.

### Mouse model for osteoporosis

All animal experiments were approved by the Animal Ethics Committee of Qilu Hospital of Shandong University. Female C57BL/6 mice (10 weeks old) were randomly divided into four groups (6 mice per group). Ovariectomies or sham operations were performed under general anesthesia. Bilateral ovaries were removed under sterile conditions. In the sham group, only part of the adipose tissue around the ovary was removed. The lentiviral intramedullary injection was administered 4 weeks after ovariectomy. As previously described [46], a 5-mm longitudinal incision was made along the medial side of the quadriceps femoris patella complex. Lateral dislocation of the patella was performed to expose the intercondylar sulcus. A fine Kirschner wire was drilled in, a 26-gauge needle was inserted, and 15 μL of WTAP overexpression lentivirus (5 × 10^7^/mL) was injected into the medullary cavity. The quadriceps femoris patella complex was then resutured. In the same way, 15 μL of lentiviral vector (5 × 10^7^/mL) was injected into the femurs of mice in the osteoporosis group. All mice were sacrificed two months later, and BMSCs were isolated from three random mice from each group. BMSCs undergo osteogenic and adipogenic differentiation under certain conditions. Micro-CT scanning and histological analysis were performed on all the mouse samples.

### MicroRNA mimic, inhibitor, and lentivirus/siRNA transfection

The miRNA mimics, miRNA inhibitors, and siRNAs (GenePharma Co., China) were used for gene overexpression or knockdown. The siRNA sequences are shown in Supplementary Table 1. Before transfection, the BMSCs culture media was removed, and fresh serum-free media (Gibco) was added. For miRNA mimic, miRNA inhibitor, siRNA, or negative control transfection, Lipofectamine 2000 transfection agent (Invitrogen, USA) was used, following the manufacturer’s instructions. Lentivirus transfection [negative control (Vector), WTAP overexpression lentivirus (OE-WTAP), negative control (shNC), and WTAP-knockdown lentivirus (shWTAP)] was performed at a 30–50% cell density. The culture medium was replaced 6–12 h after transfection and every 3 days until the cells reached 80–90% confluency. After 72 h of transfection, stably transfected cell lines were selected using 2 μg/mL puromycin.

### RNA extraction and real-time quantitative polymerase chain reaction

TRIzol (Invitrogen, USA) was used to extract RNA from the tissues or cells. cDNA was synthesized from the extracted mRNA using the Prime Script RT Reagent Kit (Takara Bio Inc., Japan) according to the standard procedure. SYBR Premix Ex Taq was used for quantitative real-time reverse transcription polymerase chain reaction (qRT-PCR). A Light Cycler 480 System (Roche Applied Science, Germany) was used for the qualitative analysis. The allele-specific primers used in this study are listed in Supplementary Table 1.

### Western blotting (WB) and co-immunoprecipitation (co-IP)

Before protein extraction, the cells were washed twice with phosphate-buffered saline (PBS). Total proteins of specific samples were extracted on ice using a protein extraction kit (BestBio, China) following the manufacturer’s instructions. The protein concentration was determined using a BCA Protein Assay Kit (Beyotime, China). The proteins were mixed with 5× SDS–PAGE loading buffer (Beyotime, China) and boiled for 5–10 min. Proteins were resolved using the PAGE Gel Fast Preparation Kit (Epizyme, USA) and transferred to polyvinylidene fluoride (PVDF) membranes (Millipore Sigma, USA). The primary antibodies used for western blot (WB) analysis are listed in Supplementary Table 2. All antibodies were diluted according to the manufacturer’s instructions.

For co-IP, whole-cell extracts were prepared in a lysis buffer after specific treatment. The extracts were incubated with the corresponding antibodies overnight at 4 °C. Protein A&G beads (Invitrogen) were added and incubated for 4 h at 4 °C, followed by washing and elution with SDS-loading buffer at 95 °C for 5 min. The eluted proteins were then identified by WB.

### Alkaline phosphatase (ALP) activity assay

Alkaline phosphatase activity was determined using the alkaline phosphatase detection kit (Beyotime, China) following the manufacturer’s protocol. Briefly, cells were lysed with cell lysis buffer (not containing phosphatase inhibitors), and the supernatant was centrifuged for alkaline phosphatase detection. The absorbance of the supernatant at 405 nm was measured using a spectrophotometer (Bio-Rad). The alkaline phosphatase activity of the samples was calculated based on the absorbance of the blank control, standard, and sample.

### Alkaline phosphatase staining

A BCIP/NBT alkaline phosphatase color development kit (Beyotime, China) was used for alkaline phosphatase staining. The staining solution was prepared by adding BCIP, NBT, and working solutions according to the recommended procedures. The mixture was then incubated for 60 min at room temperature in the dark. The reaction was terminated by removing the staining solution and washing it with water 1–2 times.

### Alizarin red staining (ARS)

An Alizarin red staining kit (Servicebio, China) was used to assess the amount of calcium salt deposits following the recommended procedures.

### Micro-CT

Mice were anesthetized and sacrificed. Femurs were dissected and fixed with 4% paraformaldehyde. A high-resolution micro-CT scanner (PerkinElmer, Japan) was used to scan the tissue. The parameters were adjusted to a voltage of 90 kV, 88 μA, and a resolution of 7 μm per pixel. Skyscan NRecon software (PerkinElmer, Japan) was used to reconstruct the images, and CTVox software (PerkinElmer, Japan) was used to analyze the parameters of the samples.

### Bone histomorphometry and immunohistochemistry

Mice were anesthetized and sacrificed. Tissues were fixed in 4% paraformaldehyde for 24 h and decalcified with EDTA for 2 weeks. H&E staining was performed according to recommended procedures. After deparaffinization and hydration, the sections were stained with hematoxylin for 5 min and 1% eosin Y for 10 min. Masson’s trichrome staining kit was used following the procedures to stain collagen fibers. Fluorochrome double labeling was performed as previously described. A double calcein (25 mg/kg) label was injected subcutaneously 10 and 3 days before the necropsy. Non-decalcified bone specimens of femurs were prepared, and the double calcein label was visualized using a microscope (Olympus, Japan) equipped with a digital camera (Olympus, Japan).

For IHC analysis, the deparaffinized sections were incubated with 3% H_2_O_2_ for 15 min and then treated with 5% BSA for 10 min. The sections were then incubated with primary antibodies overnight at 4 °C. This was followed by incubation with biotin-conjugated secondary antibodies and visualization with the streptavidin–biotin staining technique. Nuclei were stained with hematoxylin, and the slides were photographed using a microscope (ZEISS, AXIO). For all stains, samples with inadequate staining due to technical problems were excluded.

### Dual-luciferase reporter assay

A dual-luciferase reporter assay was used to verify the interaction between pri-miR-181a, pri-miR-181c, and WTAP. A luciferase vector was constructed containing either the wild-type pri-miR-181a, pri-miR-181c, or a mutant sequence. BMSCs were co-transfected with the luciferase vector with or without WTAP overexpression or knockdown. To verify the interaction between miRNA and SFRP1 mRNA, a luciferase vector containing either the wild-type 3’ untranslated region or a mutant sequence was used. BMSCs were co-transfected with the luciferase vector and miRNA mimics or their control using Lipofectamine 2000 (Invitrogen, USA). After 24 h, firefly (Fluc) and Renilla (Rluc) luciferase activities were analyzed using the Luciferase Reporter Assay System (Promega, USA). Relative luciferase activity was calculated by dividing Fluc by Rluc and normalized to the individual control for each assay.

### RNA m^6^A quantification

Total RNA was extracted using the TRIzol reagent (Invitrogen, Carlsbad, CA, USA). The EpiQuik m^6^A RNA Methylation Quantification kit (colorimetric) (EpiGentek, USA) was used to measure the relative m^6^A content following the recommended procedures. Absorbance was measured at 450 nm using a microplate reader.

### RNA immunoprecipitation (RIP) assay

The RIP kit (BersinBio, Guangzhou, China) was used according to recommended procedures. BMSCs were collected and lysed in radioimmunoprecipitation (RIPA) lysis buffer. Magnetic beads were then incubated with 10 μg antibodies and normal IgG (Millipore, MA, USA) overnight at 4 °C. Immunoprecipitated RNAs were extracted using TRIzol, and RNA enrichment was analyzed using qRT-PCR.

### RNA pull-down

Fragments of pri-miR-181a, pri-miR-181c, pri-miR-181a-AS, and pri-miR-181c-AS were amplified using primers containing T7 and SP6 promoter sequences(BioSune, Shanghai, China). The templates were transcribed using a MAXIscript™ SP6/T7 Transcription Kit in vitro (Thermo Fisher Scientific, MA, USA). N6-methyl-ATP (m^6^A) (Biorbit, orb65363) was used instead of ATP in the in vitro transcription reaction to achieve pri-miR-181a-m^6^A and pri-miR-181c-m^6^A. BMSCs (1.5 × 10^7^ cells) were collected and lysed. A Pierce™ Magnetic RNA-Protein Pull-Down Kit was used to complete the RNA pull-down experiment. RNA was first labeled with the end of the desulfurizing biotin. The labeled RNA was captured onto microbeads for 60 min at room temperature to prepare them for protein binding. The RNA-bound beads were equilibrated in protein RNA-binding buffer, and then the protein lysates were added to incubate together overnight at 4 °C. After washing with ice-cold PBS three times, the samples were eluted using sodium dodecyl sulfate–polyacrylamide gel electrophoresis loading buffer. Eluted samples were prepared for WB.

### Statistical analysis

All data are shown as mean ± standard deviation (SD). All experiments were repeated at least three times. The Student’s *t-*test was used for comparisons between two independent groups, and one-way analysis of variance (ANOVA) was used for comparisons between multiple groups. Statistical significance was set at *P* < 0.05.

## Supplementary information

Supplemental Material

Original Data File

reproducibility checklist

## Data Availability

The datasets used and/or analyzed during the current study are available from the corresponding author on reasonable request.
