# Supplementary material for: WTAP-mediated m6A modification modulates bone marrow mesenchymal stem cells differentiation potential and osteoporosis
Source: Cell Death Dis. 2023 Jan 17;14(1):33. doi: 10.1038/s41419-023-05565-x (PMC9845239; doi:10.1038/s41419-023-05565-x)
Supplement: Supplementary file 1 — Supplemental Material [file 41419_2023_5565_MOESM1_ESM.pdf]

WTAP-mediated m6A modification modulates bone marrow mesenchymal stem cells differentiation potential and osteoporosis

Supplementary materials

Supplementary Figure

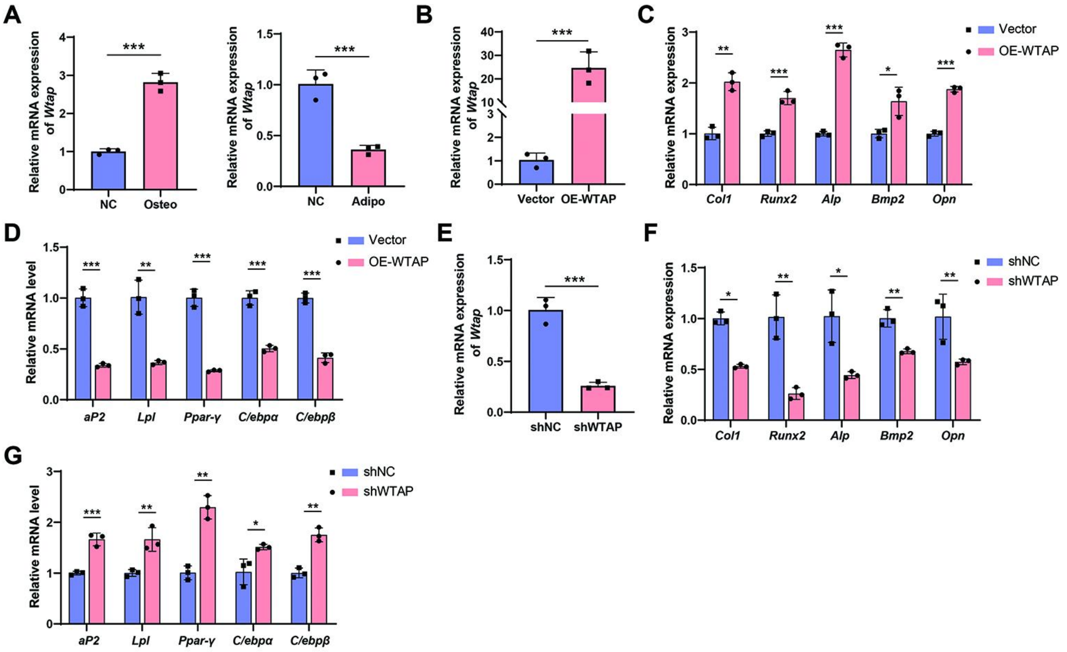

Supplementary Figure Legends

Supplementary figure. 1 (A) The mRNA expression of WTAP on day 3 during osteogenic differentiation or adipogenic differentiation. **(B)** The mRNA expression of WTAP in the OE-WTAP primary mouse BMSCs and control BMSCs. **(C)** The mRNA expression of osteogenic-related genes on day 3 during osteogenic differentiation in the OE-WTAP primary mouse BMSCs and Vector BMSCs. **(D)** The mRNA expression of adipogenic-related genes on day 3 during adipogenic differentiation in the OE-WTAP primary mouse BMSCs and Vector BMSCs. **(E)** The mRNA expression of WTAP in the BMSCs with shWTAP or shNC. **(F)** The mRNA expression of osteogenic-related genes of BMSCs with shNC and shWTAP. **(G)** The mRNA expression of adipogenic-related genes of BMSCs with shNC and shWTAP. Data are expressed as the mean ±SD, *p <0.05, **p <0.01, ***p <0.001.

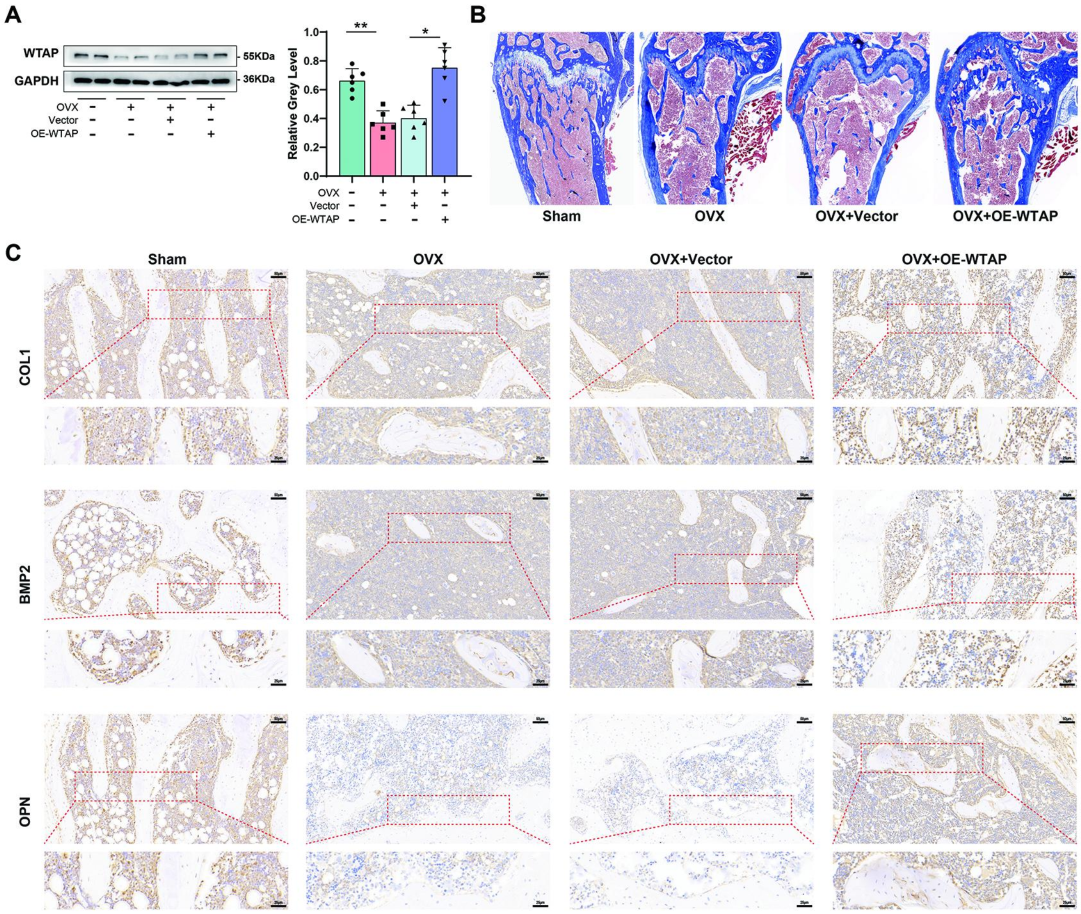

Supplementary figure. 2 (A) The protein expression of WTAP in primary BMSCs obtained from different mice group after 2 months. **(B)** Representative Masson's staining of femurs in different mice group. **(C)** Representative IHC images of COL1, BMP and OPN in the femur sections. Data are expressed as the mean ±SD, *p <0.05, **p <0.01.

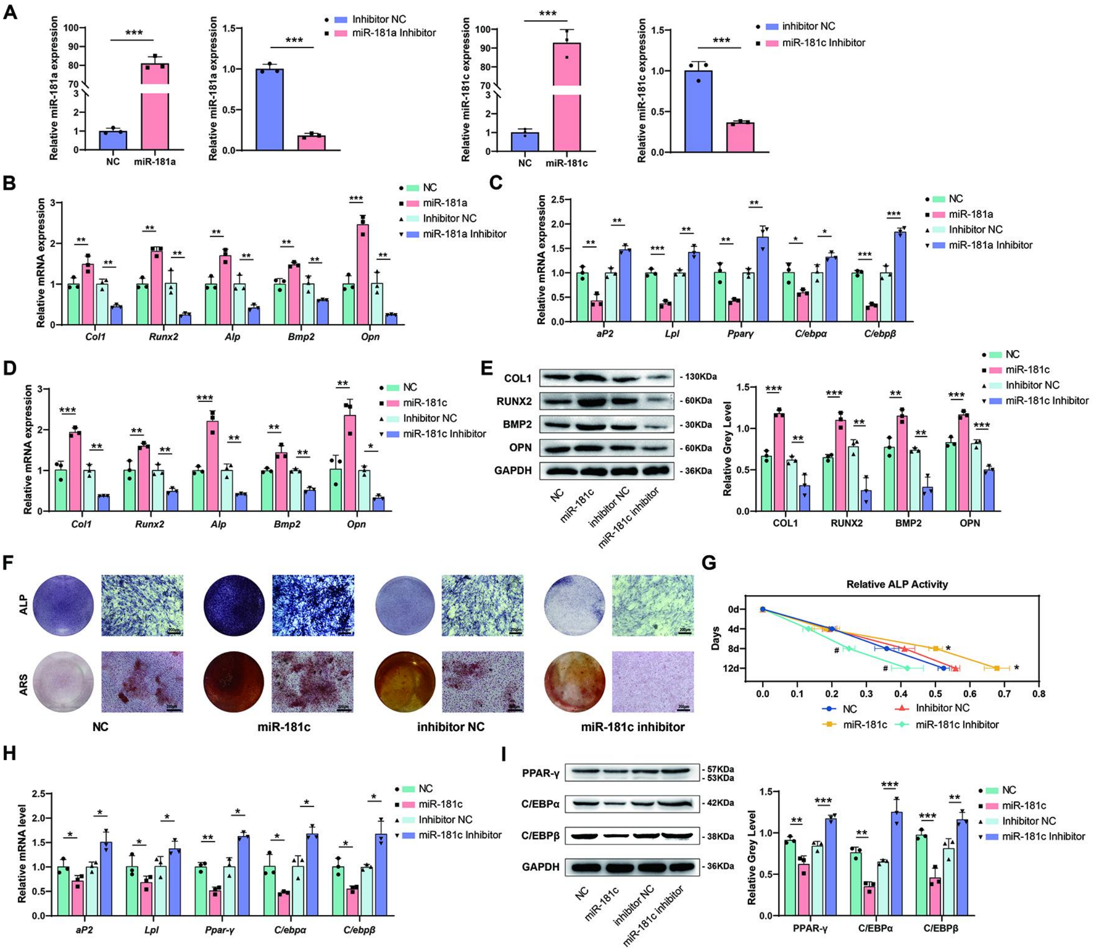

Supplementary figure. 3 (A) The relative miRNA expression of BMSCs treated with mimics or inhibitors. **(B)** The mRNA expression of osteogenic-related genes in primary BMSCs treated with NC, miR-181a, inhibitor NC, miR-181a inhibitor on day 3 during osteogenic induction. **(C)** The mRNA or protein expression of adipogenic-related genes in primary BMSCs treated with NC, miR-181a, inhibitor NC, miR-181a inhibitor on day 3 during adipogenic induction. **(D-E)** The mRNA or protein expression of osteogenic-related genes in primary BMSCs treated with NC, miR-181C, inhibitor NC, miR-181c inhibitor on day 3 during osteogenic induction. **(F-G)** ALP staining on day 7, ARS staining on day 14 and relative ALP activity of primary BMSCs treated with NC, miR-181c, inhibitor NC, miR-181c inhibitor during osteogenic induction. **(H-I)** The mRNA or protein expression of adipogenic-related genes in primary BMSCs treated with NC, miR-181c, inhibitor NC, miR-181c inhibitor on day 3 during adipogenic induction. Data are expressed as the mean ±SD, *p <0.05, **p <0.01, ***p <0.001.

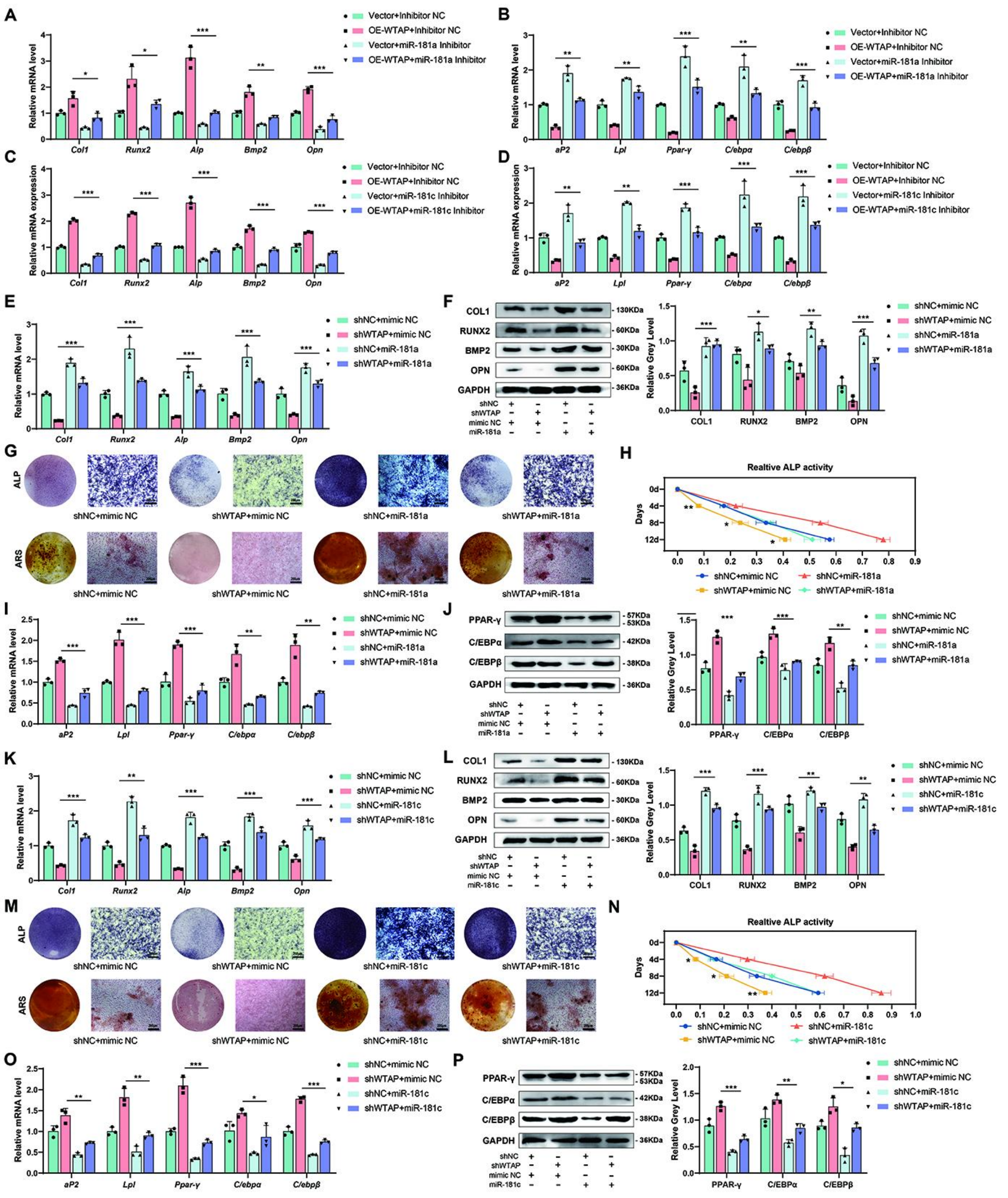

Supplementary figure. 4 (A) The mRNA expression of osteogenic-related genes in primary BMSCs (Vector+inhibitor NC; OE-WTAP+inhibitor NC; Vector+miR-181a inhibitor; OE-WTAP+miR-181a inhibitor) on day 3 during osteogenic induction. **(B)** The mRNA expression of adipogenic-related genes in primary BMSCs (Vector+inhibitor NC; OE-WTAP+inhibitor NC; Vector+miR-181a inhibitor; OE-WTAP+miR-181a inhibitor) on day 3 during adipogenic induction. **(C)** The mRNA expression of osteogenic-related genes in primary BMSCs (Vector+inhibitor NC; OE-WTAP+inhibitor NC; Vector+miR-181c inhibitor; OE-WTAP+miR-181c inhibitor) on day 3 during osteogenic induction. **(D)** The mRNA expression of adipogenic-related genes in primary BMSCs (Vector+inhibitor NC; OE-WTAP+inhibitor NC; Vector+miR-181c inhibitor; OE-WTAP+miR-181c inhibitor) on day 3 during adipogenic induction. **(E-F)** The mRNA or protein expression of osteogenic-related genes in primary BMSCs (sh-NC+mimic NC; sh-WTAP+mimic NC; sh-NC+miR-181a; sh-WTAP+miR-181a) on day 3

during osteogenic induction. **(G-H)** ALP staining on day 7, ARS staining on day 14 and relative ALP activity of primary BMSCs (sh-NC+mimic NC; sh-WTAP+mimic NC; sh-NC+miR-181a; sh-WTAP+miR-181a) during osteogenic induction. **(I-J)** The mRNA or protein expression of adipogenic-related genes in primary BMSCs (sh-NC+mimic NC; sh-WTAP+mimic NC; sh-NC+miR-181a; sh-WTAP+miR-181a) on day 3 during adipogenic induction. **(K-L)** The mRNA or protein expression of osteogenic-related genes in primary BMSCs (sh-NC+mimic NC; sh-WTAP+mimic NC; sh-NC+miR-181c; sh-WTAP+miR-181c) on day 3 during osteogenic induction. **(M-N)** ALP staining on day 7, ARS staining on day 14 and relative ALP activity of primary BMSCs (sh-NC+mimic NC; sh-WTAP+mimic NC; sh-NC+miR-181c; sh-WTAP+miR-181c) during osteogenic induction. **(O-P)** The mRNA or protein expression of adipogenic-related genes in primary BMSCs (sh-NC+mimic NC; sh-WTAP+mimic NC; sh-NC+miR-181c; sh-WTAP+miR-181c) on day 3 during adipogenic induction. Data are expressed as the mean ±SD, *p <0.05, **p <0.01, ***p <0.001.

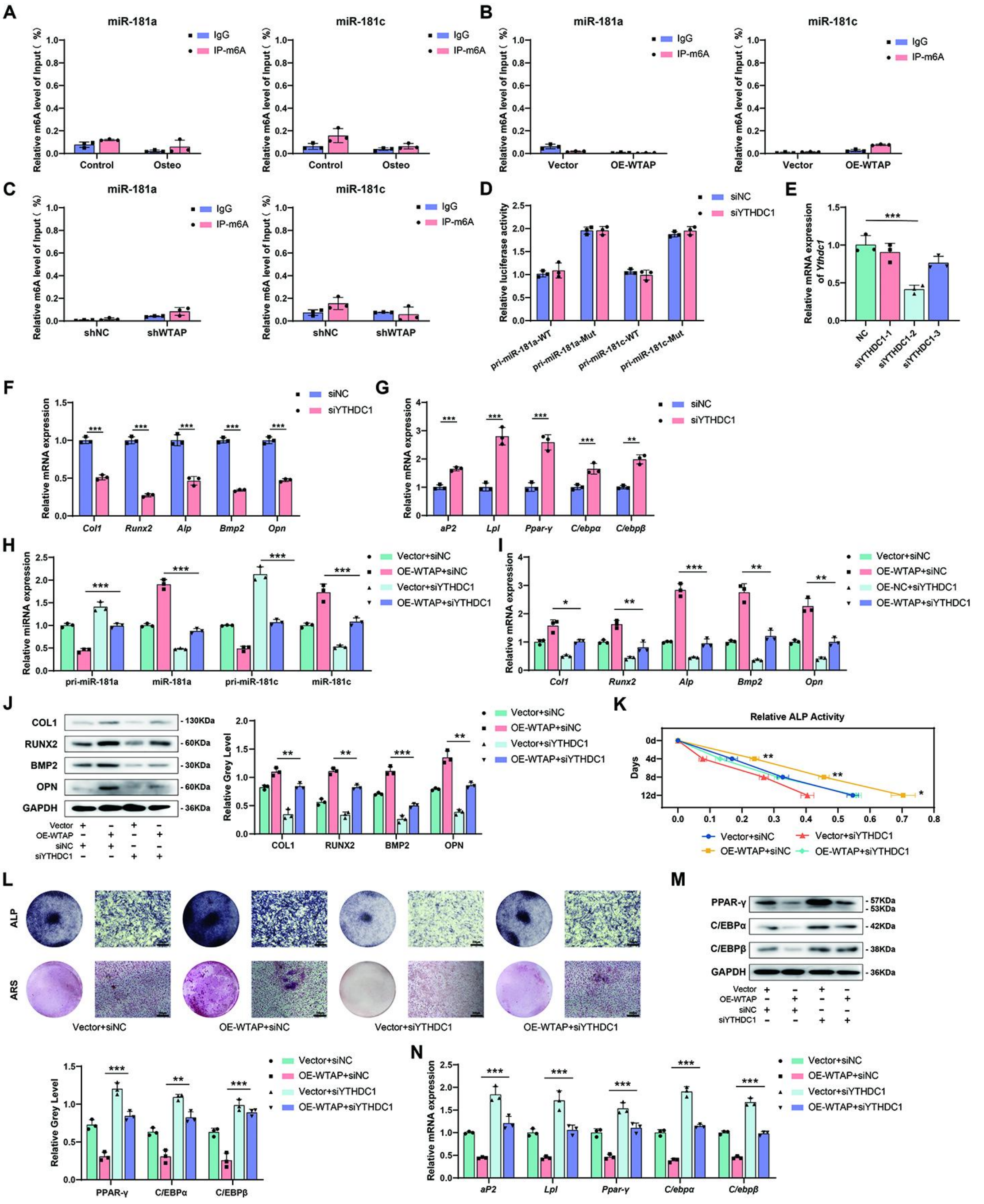

Supplementary figure. 5 (A) m6A level of miR-181a and miR-181c in BMSCs during osteogenic differentiation by Me-RIP-qPCR. **(B)** m6A level of miR-181a and miR-181c in Vector or OE-WTAP BMSCs by Me-RIP-qPCR. **(C)** m6A level of miR-181a and miR-181c in sh-NC or sh-WTAP BMSCs by Me-RIP-qPCR. **(D)** Dual-luciferase reporter assay of wild-type or site-mutant BMSCs with YTHDC1 knockdown or not. **(E)** The mRNA level of YTHDC1 of BMSCs with siYTHDC1 or siNC. **(F)** The mRNA expression of osteogenic-related genes in primary BMSCs with siNC or siYTHDC1 on day 3 during osteogenic induction. **(G)** The mRNA expression of adipogenic-related genes in primary BMSCs with siNC or siYTHDC1 on day 3 during adipogenic induction. **(H)** The relative expression of pri-miR-181a, pri-miR-181c, miR-181a and miR-181c in primary BMSCs (Vector+siNC; OE-WTAP+siNC; Vector+siYTHDC1;

OE-WTAP+siYTHDC1). **(I-J)** The mRNA or protein expression of osteogenic-related genes in primary BMSCs (Vector+siNC; OE-WTAP+siNC; Vector+siYTHDC1; OE-WTAP+siYTHDC1) on day 3 during osteogenic induction. **(K-L)** Relative ALP activity, ALP staining on day 7 and ARS staining on day 14 of primary BMSCs (Vector+siNC; OE-WTAP+siNC; Vector+siYTHDC1; OE-WTAP+siYTHDC1) during osteogenic induction. **(M-N)** The mRNA or protein expression of adipogenic-related genes in primary BMSCs (Vector+siNC; OE-WTAP+siNC; Vector+siYTHDC1; OE-WTAP+siYTHDC1) on day 3 during adipogenic induction. Data are expressed as the mean ±SD, *p <0.05, **p <0.01, ***p <0.001.

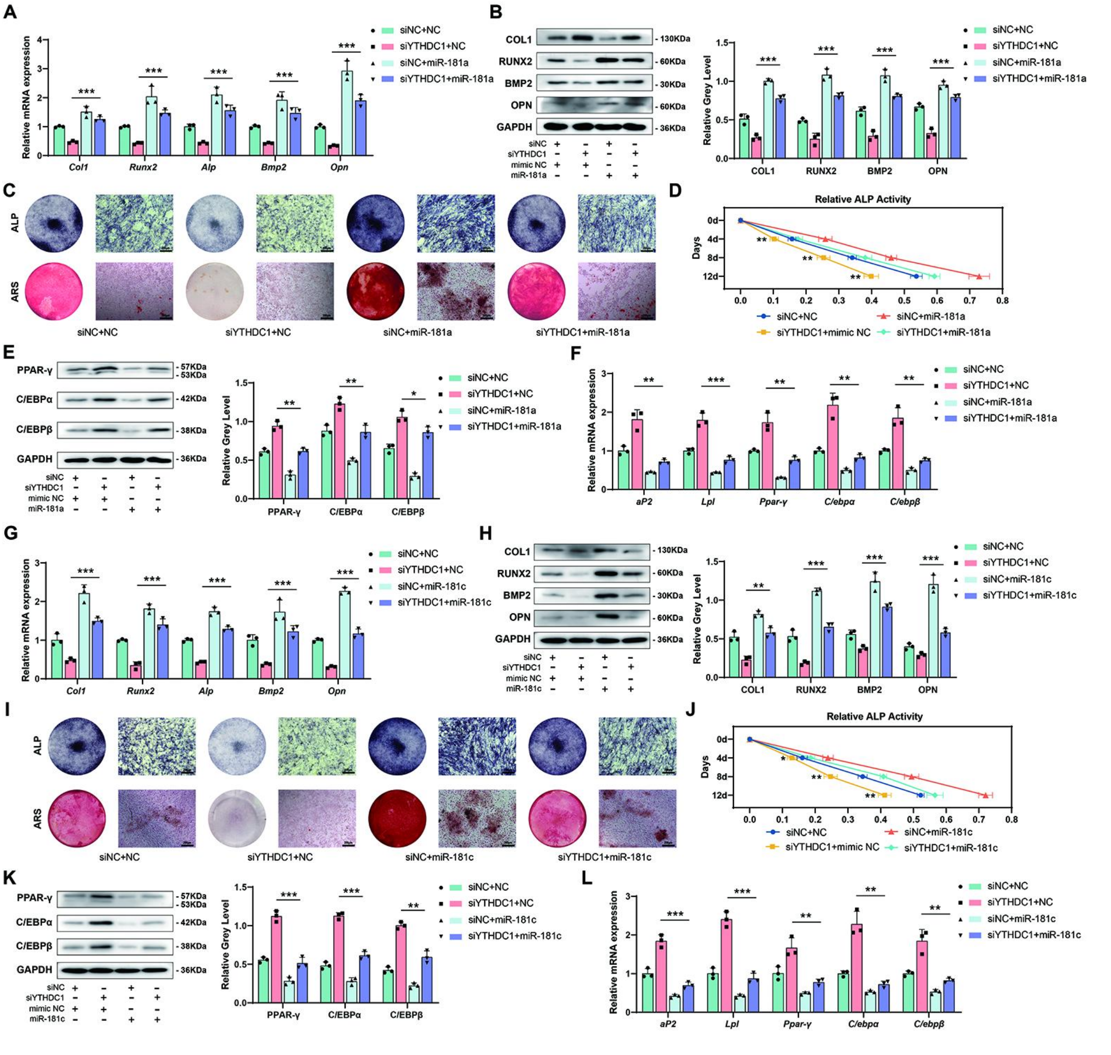

Supplementary figure. 6 (A-B) The mRNA or protein expression of osteogenic-related genes in primary BMSCs (siNC+NC; siYTHDC1+NC; siNC+miR-181a; siYTHDC1+miR-181a) on day 3 during osteogenic induction. (**C-D)** ALP staining on day 7, ARS staining on day 14 and relative ALP activity of primary BMSCs (siNC+NC; siYTHDC1+NC; siNC+miR-181a; siYTHDC1+miR-181a) during osteogenic induction. **(E-F)** The mRNA or protein expression of adipogenic-related genes in primary BMSCs (siNC+NC; siYTHDC1+NC; siNC+miR-181a; siYTHDC1+miR-181a) on day 3 during adipogenic induction. **(G-H)** The mRNA or protein expression of osteogenic-related genes in primary BMSCs (siNC+NC; siYTHDC1+NC; siNC+miR-181c; siYTHDC1+miR-181c) on day 3 during osteogenic induction. **(I-J)** ALP staining on day 7, ARS staining on day 14 and relative ALP activity of primary BMSCs

(siNC+NC; siYTHDC1+NC; siNC+miR-181c; siYTHDC1+miR-181c) during osteogenic induction. **(K-L)** The mRNA or protein expression of adipogenic-related genes in primary BMSCs (siNC+NC; siYTHDC1+NC; siNC+miR-181c; siYTHDC1+miR-181c) on day 3 during adipogenic induction. Data are expressed as the mean ±SD, *p <0.05, **p <0.01, ***p <0.001.

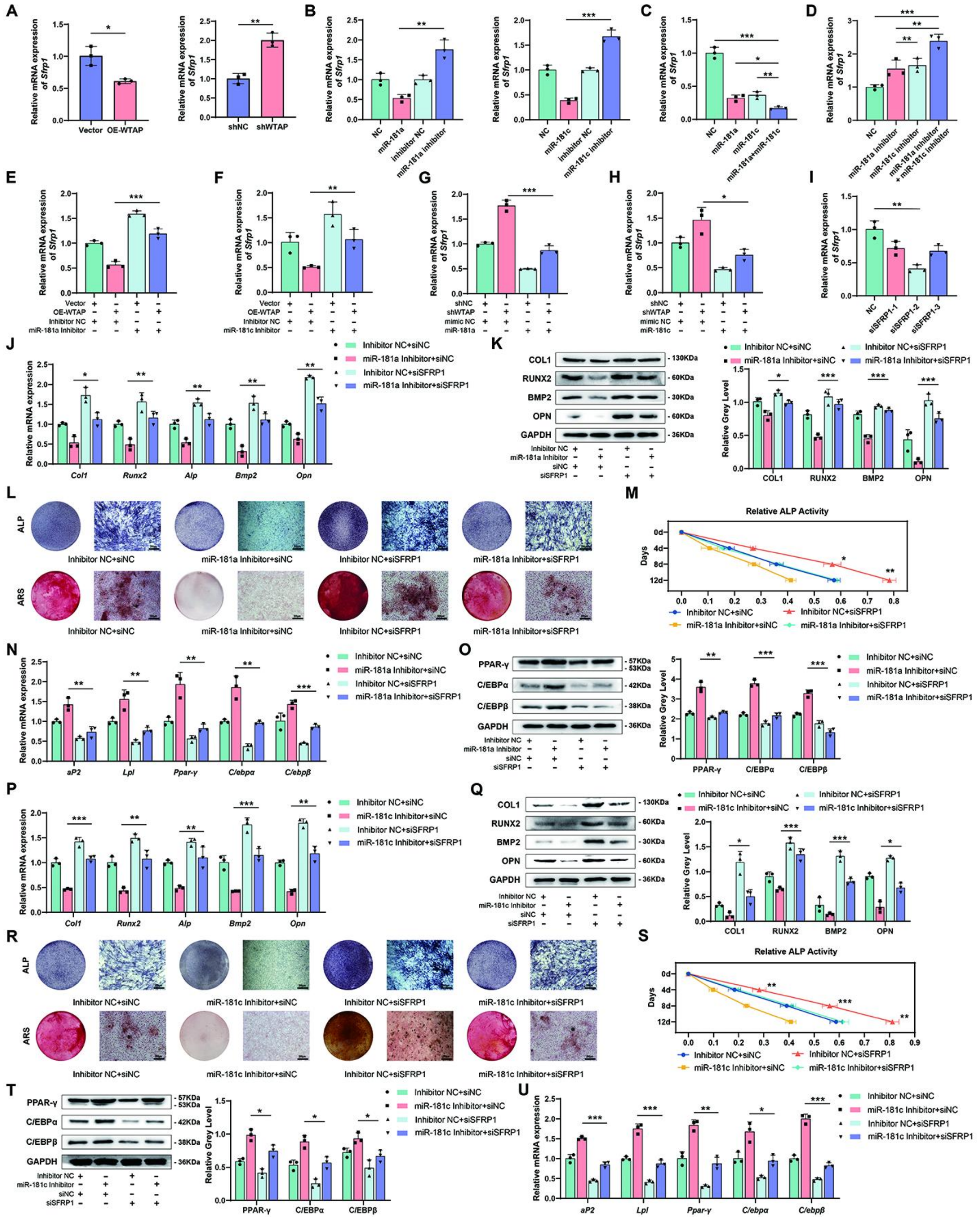

Supplementary figure. 7 (A) The mRNA expression of SFRP1 in BMSCs with WTAP overexpression or knockdown. **(B)** The mRNA expression of SFRP1 in BMSCs with miR-181a, miR-181a inhibitor or miR-181c, miR-181c inhibitor. **(C)** The mRNA expression of SFRP1 in BMSCs with miR-181a, miR-181c or miR-181a and miR-181c. **(D)** The mRNA expression of SFRP1 in BMSCs with miR-181a inhibitor, miR-181c inhibitor or miR-181a inhibitor and miR-181c inhibitor. **(E-H)** The mRNA expression of SFRP1 in BMSCs with WTAP and miRNA manipulation simultaneously. **(I)** The mRNA level of SFRP1 of BMSCs with siSFRP1 or siNC.

(J-K) The mRNA or protein expression of osteogenic-related genes in primary BMSCs (inhibitor NC+siNC; miR-181a inhibitor+siNC; inhibitor NC+siSFRP1; miR-181a inhibitor+siSFRP1) on day 3 during osteogenic induction. **(L-M)** ALP staining on day 7, ARS staining on day 14 and relative ALP activity of primary BMSCs (inhibitor NC+siNC; miR-181a inhibitor+siNC; inhibitor NC+siSFRP1; miR-181a inhibitor+siSFRP1) during osteogenic induction. **(N-O)** The mRNA or protein expression of adipogenic-related genes in primary BMSCs (inhibitor NC+siNC; miR-181a inhibitor+siNC; inhibitor NC+siSFRP1; miR-181a inhibitor+siSFRP1) on day 3 during adipogenic induction. **(P-Q)** The mRNA or protein expression of osteogenic-related genes in primary BMSCs (inhibitor NC+siNC; miR-181c inhibitor+siNC; inhibitor NC+siSFRP1; miR-181c inhibitor+siSFRP1) on day 3 during osteogenic induction. (**R-S)** ALP staining on day 7, ARS staining on day 14 and relative ALP activity of primary BMSCs (inhibitor NC+siNC; miR-181c inhibitor+siNC; inhibitor NC+siSFRP1; miR-181c inhibitor+siSFRP1) during osteogenic induction. **(T-U)** The mRNA or protein expression of adipogenic-related genes in primary BMSCs (inhibitor NC+siNC; miR-181c inhibitor+siNC; inhibitor NC+siSFRP1; miR-181c inhibitor+siSFRP1) on day 3 during adipogenic induction. Data are expressed as the mean ±SD, *p <0.05, **p <0.01, ***p <0.001.

Supplementary Table 1. The sequences of siRNAs and primers used this study.

Sequence of primers used for real-time PCR

gene	sense	antisense
h-METTL3	CTTTCTACCCCATCTTGAGTG	CCAACCTTCCGTAGTGATAGTC
h-METTL14	GAACACAGAGCTTAAATCCCCA	TGTCAGCTAAACCTACATCCCTG
h-WTAP	TTCCCAAGAAGGTTCGATTG	TGCAGACTCCTGCTGTTGTT
h-FTO	GACTCGTCCTCACTTTCATCC	AAGAGCAGAGCAGCCTACAAC
h-ALKBH5	GTGGGACCTTTTGGGTTTCAG	GCATACGGCCTCAGGACATTA
h-GAPDH	GGAGCGAGATCCCTCCAAAAT	GGCTGTTGTCATACTTCTCATGG
m-WTAP	GGCGAAGTGTCGAATGCT	CCAACTGCTGGCGTGTCT
m-GAPDH	AAATCCCATCACCATCTTCCAG	AGGGGCCATCCACAGTCTTCT
m-COL1a1	CCCTGGTCCCTCTGGAAATG	GGACCTTTGCCCCCTTCTTT
m-RUNX2	GGGACTGTGGTTACCGTCAT	ATAACAGCGGAGGCATTTCG
m-ALP	GCACCTGCCTTACCAACTCT	GTGGAGACGCCCATACCATC
m-BMP2	GGAAAACTTCCCGACGCTTCT	CCTGCATTTGTTCCCGAAAA
m-OPN	CACATGAAGAGCGGTGAGTCT	CCCTTTCCGTTGTTGTCCTG
m-LPL	ACAAGAGAGAACCAGACTCCAA	AGGGTAGTTAAACTCCTCCTCC
m-αP2	AGCACCATAACCTTAGATGGGG	CGTGGAAGTGACGCCTTTCA
m-PPARγ	GCCGAGTCTGTGGGGATAAA	TCCGGCAGTTAAGATCACACC
m-C/EBPα	AGGAACACGAAGCACGATCAG	CGCACATTCACATTGCACAA
m-C/EBPβ	CTTCAGCCCGTACCTGGAG	GGAGAGGAAGTCGTGGTGC
h-miR-221	AGCUACAUUGUCUGCUGGGUUUC	
h-miR-200c	UAAUACUGCCGGGUAAUGAUGGA	
h-miR-25	CAUUGCACUUGUCUCGGUCUGA	
h-miR-378c	ACUGGACUUGGAGUCAGAAGAGUGG	
h-miR-574	CACGCUCAUGCACACACCCACA	
h-miR-181a	AACAUUCAACGCUGUCGGUGAGU	
h-miR-181c	AACAUUCAACCUGUCGGUGAGU	
h-U6	CAGCACATATACTAAAATTGGAACG	ACGAATTTGCGTGTCATCC
m-miR-181a	TTCCACCACTGACCGTTGACT	TATCCTTGTTCACGACTCCTTCAC
m-miR-181c	AGCAACATTCAACCTGTCGG	AGAGCAGGGTCCGAGGTA
m-pri-miR-181a	CACCGACCGTTGACTGTACC	AAGAGGCCACAGGACTGTAT
m-pri-miR-181c	AGCUACAUUGUCUGCUGGGUUUC	AGCCCATCAAGATCCCAGAC
m-U6	CAGCACATATACTAAAATTGGAACG	ACGAATTTGCGTGTCATCC
m-YTHDC1	GCAAGACUUUCUUCAGAAUTT	AUUCUGAAGAAAGUCUUGCTT
m-YTHDC2	GCGACUCAACAAUGGCAUATT	UAUGCCAUUGUUGAGUCGCTT
m-YTHDF1	ATGACAATGACTTTGAGCCCTA	AGGGAGTAAGGAAATCCAATGG
m-YTHDF2	ACTTCTCAGCATGGGGAAATAA	TATTCATGCCAGGAGCCTTATT
m-YTHDF3	GCTCCACCAACCCAACCAGTTC	CTGAGGTCCTTGTTGCTGCTGTG
m-YTHDC1	AGTGACTCTGGTTCTGAATCTG	CTGGTTTGATCTTTTCGGACAG
m-YTHDC2	GAGAATTGGGCTGTCGTTAAAG	TGAAGCAGGATGAAATCGTACT
m-IGF2BP1	GATGAAGGCCATCGAAACTTTC	GGGGTGGAATATTTCGGATTTG
m-IGF2BP2	GATGAACAAGCTTTACATCGGG	GATTTTCCCATGCAATTCCACT
m-IGF2BP3	GAGGCGCTTTCAGGTAAAATAG	AATGAGGCGGGATATTTCGTAT

h-SFRP1	CAGCGAGTTTGCACTGAGGAT	GCCCCATTCTTCAGGTACAGG
m-SFRP1	CTACTGGCCCGAGATGCTCAA	AACTCGTTGTCGCATGGAGGA

Sequences of siRNAs used this study.

gene	primer (forward)	primer (reverse）
si-NC	UUCUCCGAACGUGUCACGUTT	ACGUGACACGUUCGGAGAATT
siYTHDC1-1	GUCGGUUACAGAAUAUAAATT	GCCAGAUUGUCUUCAGAAUTT
siYTHDC1-2	GCCAGAUUGUCUUCAGAAUTT	AUUCUGAAGACAAUCUGGCTT
siYTHDC1-3	GCGUGAAUUACCCUUUACUTT	AGUAAAGGGUAAUUCACGCTT
si-NC	UUCUCCGAACGUGUCACGUTT	ACGUGACACGUUCGGAGAATT
siSFRP1-1	GGCUUGUGCUGUUCCUGAAdTdT	UUCAGGAACAGCACAAGCCdTdT
siSFRP1-2	GCUCUGUUCCUACAGCGAAdTdT	UUCGCUGUAGGAACAGAGCdTdT
siSFRP1-3	GAUGCUCAAAUGUGACAAGUUdTdT	AACUUGUCACAUUUGAGCAUCdTdT
mimics NC	UUCUCCGAACGUGUCACGUTT	ACGUGACACGUUCGGAGAATT
miR-181a mimic	ACCACCGACCGUUGACUGUACC	UACAGUCAACGGUCGGUGGUUU
miR-181c mimic	AACAUUCAACCUGUCGGUGAGU	UCACCGACAGGUUGAAUGUUUU
inhibitor NC	CAGUACUUUUGUGUAGUACAA	
miR-181a inhibitor	GGUACAGUCAACGGUCGGUGGU	
miR-181c inhibitor	ACUCACCGACAGGUUGAAUGUU	

Supplementary Table 2 List of Primary Antibodies Used In the study

primary antibodies against	company	dot
WTAP	Cell Signaling Technology	#56501S
METTL3	Abcam	ab195352
METTL14	Abclonal	A8530
COL1	Abcam	ab260043
RUNX2	Cell Signaling Technology	#12556S
BMP2	Abcam	ab214821
OPN	Abcam	ab283656
PPARγ	Cell Signaling Technology	#2435S
C/EBPα	Cell Signaling Technology	#8178S
C/EBPβ	Cell Signaling Technology	#3087S
YTHDF1	Proteintech	17479-1-AP
YTHDF2	Proteintech	24744-1-AP
YTHDF3	Abclonal	A8395
YTHDC1	Cell Signaling Technology	#77422S
YTHDC2	Abclonal	A15004
IGF2BP1	Abcam	ab290736
IGF2BP2	Abcam	ab124930
IGF2BP3	Abclonal	A4444
IgG	BersinBio	bes5101
m6A	Abcam	ab208577
GAPDH	Abclonal	AC002
SFRP1	Abcam	ab126613
DGCR8	Abcam	ab191875

Supplementary Table 3 Clinical data of bone tissue						
Normal				OP		
No.	Age	BMD of L2-L4		No.	Age	BMD of L2-L4
1	59	-0.9		1	57	-2.6
2	55	-1.2		2	62	-2.7
3	54	-1.3		3	66	-2.7
4	65	-1.3		4	69	-2.8
5	54	-1.5		5	58	-2.9
6	68	-0.8		6	66	-3.0
7	71	-1.7		7	66	-3.2
8	59	-1.7		8	66	-3.2
9	63	-1.8		9	63	-3.3
10	70	-1.9		10	67	-3.3
11	63	-0.3		11	56	-3.6
12	66	-2.1		12	63	-3.6
13	63	-2.1		13	68	-3.8
14	62	-1.5		14	69	-3.9
15	67	-2.3		15	65	-4.2
16	74	-2.3		16	73	-4.3
17	51	0.6				
18	56	0.3				

	Mean of age	SD	P value
Normal	62.2222	6.54896	0.2313
OP	64.625	4.63141	

Supplementary Table 4 Clinical data of blood						
Normal				OP		
No.	Age	BMD of L2-L4		No.	Age	BMD of L2-L4
1	59	-0.9		1	57	-2.6
2	55	-1.2		2	62	-2.7
3	54	-1.3		3	66	-2.7
4	65	-1.3		4	69	-2.8
5	54	-1.5		5	58	-2.9
6	68	-0.8		6	66	-3.0
7	71	-1.7		7	66	-3.2
8	59	-1.7		8	66	-3.2
9	63	-1.8		9	63	-3.3
10	70	-1.9		10	67	-3.3
11	63	-0.3		11	56	-3.6
12	66	-2.1		12	63	-3.6
13	63	-2.1		13	68	-3.8

14	62	-1.5
15	67	-2.3
16	74	-2.3
17	51	0.6
18	56	0.3
19	71	-0.6
20	69	2.1
21	73	-0.4
22	72	1.9
23	75	-0.9
24	70	0.4
25	72	0.2
26	83	-0.7
27	70	0.5
28	49	-0.7
29	69	-0.5
30	64	-0.6
31	64	-0.8
32	58	0.1
33	65	1.3
34	63	-0.5
35	78	-0.7
36	59	0.3
37	68	0.3
38	64	-0.5
39	82	-0.5
40	67	-0.7
41	77	0.3
42	60	0.4
43	81	-0.2
44	84	0.1
45	77	0.3
46	81	0.6
47	75	0.2
48	70	-0.7
49	76	-0.6
50	78	0.9
51	65	5.1
52	62	-0.5
53	66	-0.8
54	71	-0.5
55	81	-0.4
56	73	0.2

14	69	-3.9
15	65	-4.2
16	73	-4.3
17	53	-2.8
18	63	-2.9
19	64	-2.9
20	69	-2.9
21	62	-3.3
22	68	-3.4
23	67	-2.9
24	69	-3
25	80	-3.7
26	68	-3
27	70	-3.6
28	69	-3.4
29	62	-3.2
30	61	-3.5
31	67	-3.1
32	75	-3.6
33	75	-3.6
34	76	-3.2
35	83	-3.2
36	74	-3.1
37	70	-3.2
38	76	-3.7
39	66	-3.5
40	79	-3.4
41	61	-3.6
42	72	-2.9
43	61	-3.1
44	76	-3.5
45	59	-3.7
46	83	-2.9
47	65	-3.4
48	82	-3.7
49	68	-3
50	75	-3.3
51	63	-3.5
52	77	-2.9
53	69	-3.3
54	61	-2.9
55	75	-3
56	74	-3

57	71	4.1	57	73	-3.4
58	63	-0.5	58	69	-3
59	73	-0.8	59	65	-3.2
60	61	-0.2	60	72	-3.3
61	77	-0.1	61	91	-3.3
62	72	-0.8	62	72	-3.3
63	72	-0.5	63	69	-2.9
64	65	0.7	64	59	-3
65	63	4.3	65	68	-3.2
66	67	-0.2	66	74	-3.2
67	83	0.9	67	63	-3.4
68	70	0.6	68	67	-3.7

	Mean of age	SD	P value
Normal	68.2206	8.07769	0.787
OP	68.5735	7.07806	
