# Supplementary material for: WTAP-mediated m6A modification modulates bone marrow mesenchymal stem cells differentiation potential and osteoporosis
Source: Cell Death Dis. 2023 Jan 17;14(1):33. doi: 10.1038/s41419-023-05565-x (PMC9845239; doi:10.1038/s41419-023-05565-x)

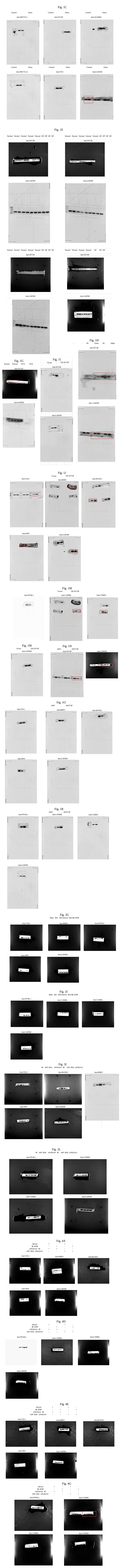
Fig. 1C
Fig. 1E
Fig. 1H
Fig. 1G
Fig. 1I
Fig. 1J
Fig. 1M
Fig. 1M
Fig. 1N
Fig. 1O
Fig. 1R
Fig. 2G
Fig. 2I
Fig. 3I
Fig. 3L
Fig. 4A
Fig. 4D
Fig. 4E
Fig. 4G

Fig. 5F

Input		IP			
		IgG		WTAP	
Control	Osteo	Control	Osteo	Control	Osteo

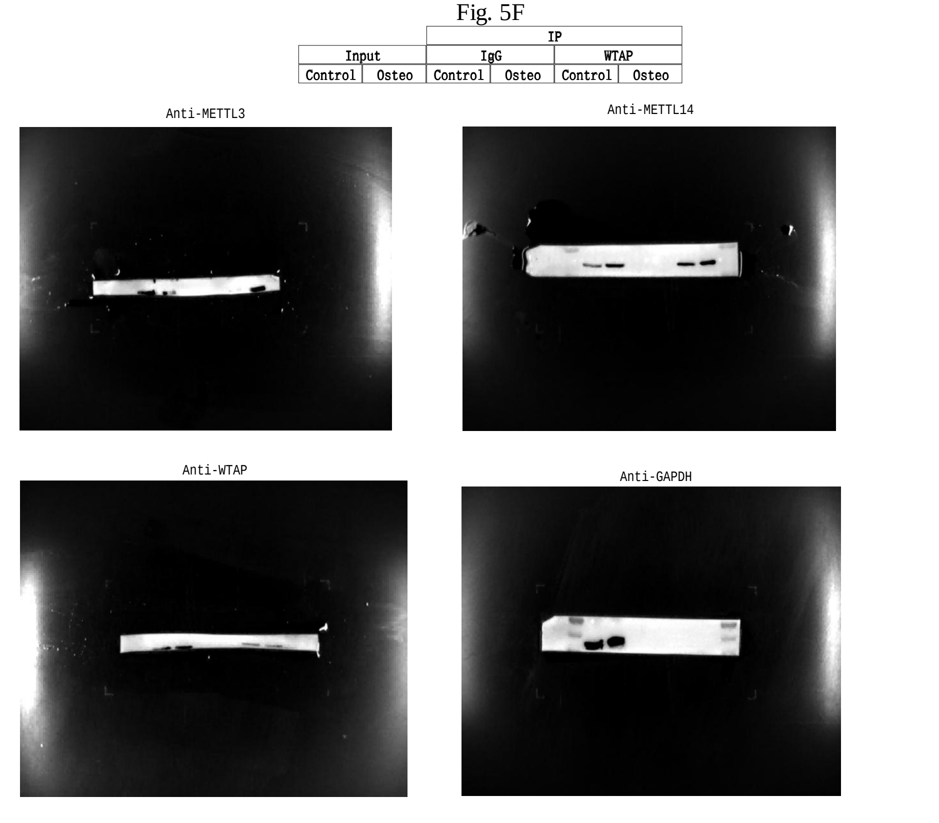

Fig. 5G

Input		pri-miR-181a-AS		pri-miR-181a		pri-miR-181c-AS		pri-miR-181a	
Control	Osteo	Control	Osteo	Control	Osteo	Control	Osteo	Control	Osteo

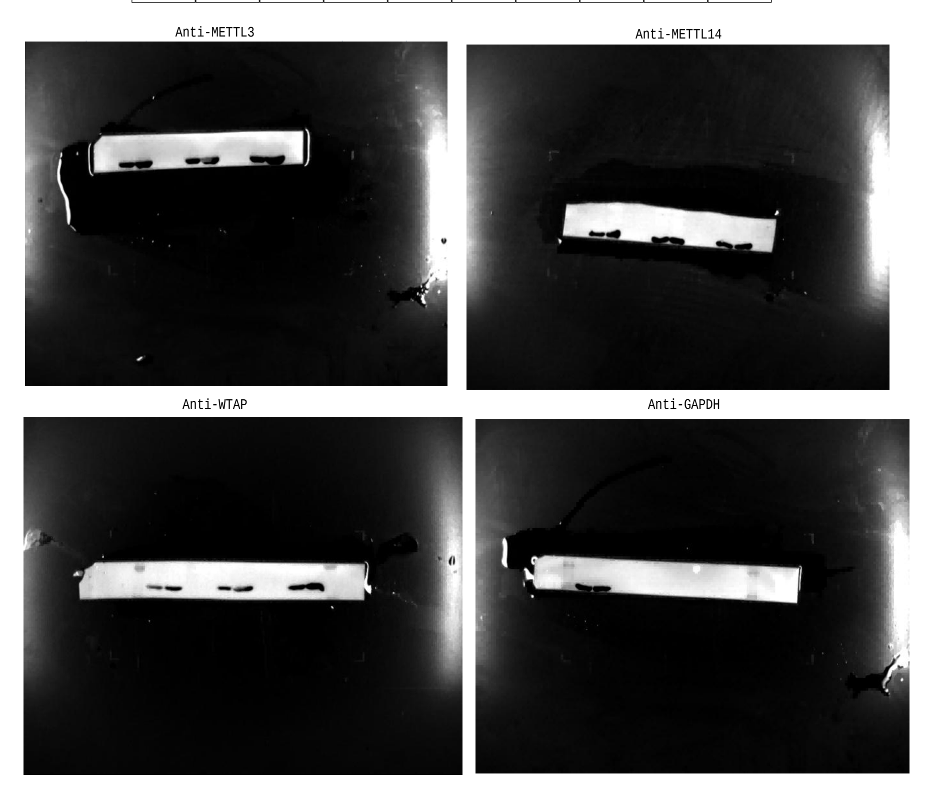

Fig. 5H

Input		pri-miR-181a-AS		pri-miR-181a		pri-miR-181c-AS		pri-miR-181a	
Vector	OE-WTAP	Vector	OE-WTAP	Vector	OE-WTAP	Vector	OE-WTAP	Vector	OE-WTAP

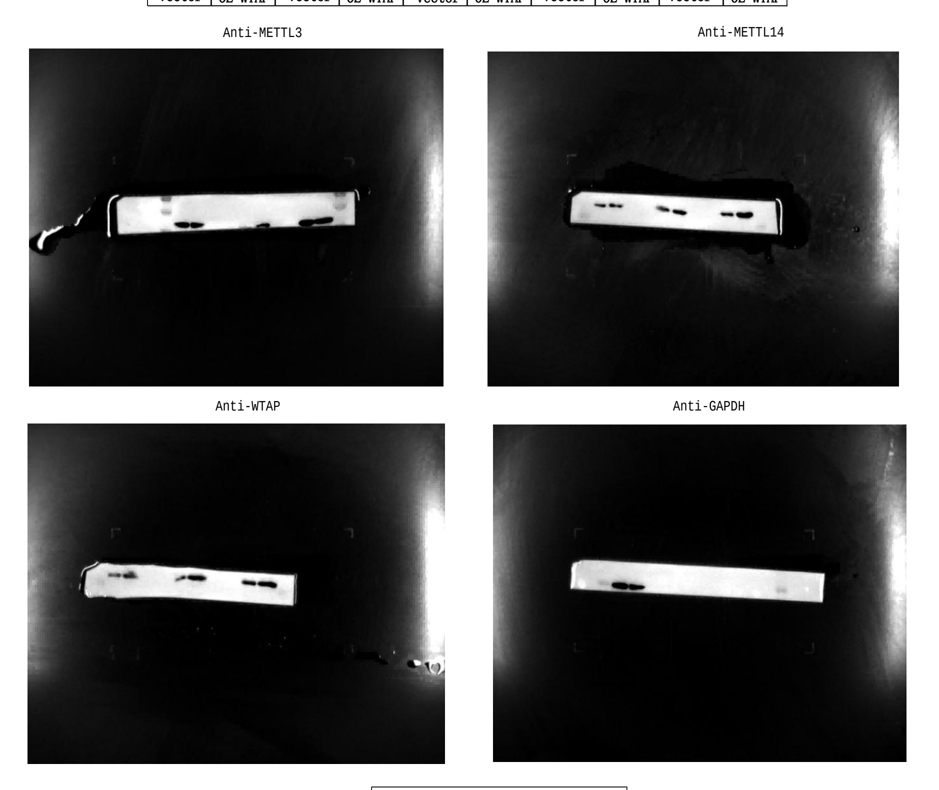

Fig. 5I

Input		pri-miR-181a-AS		pri-miR-181a		pri-miR-181c-AS		pri-miR-181a	
shNC	shWTAP	shNC	shWTAP	shNC	shWTAP	shNC	shWTAP	shNC	shWTAP

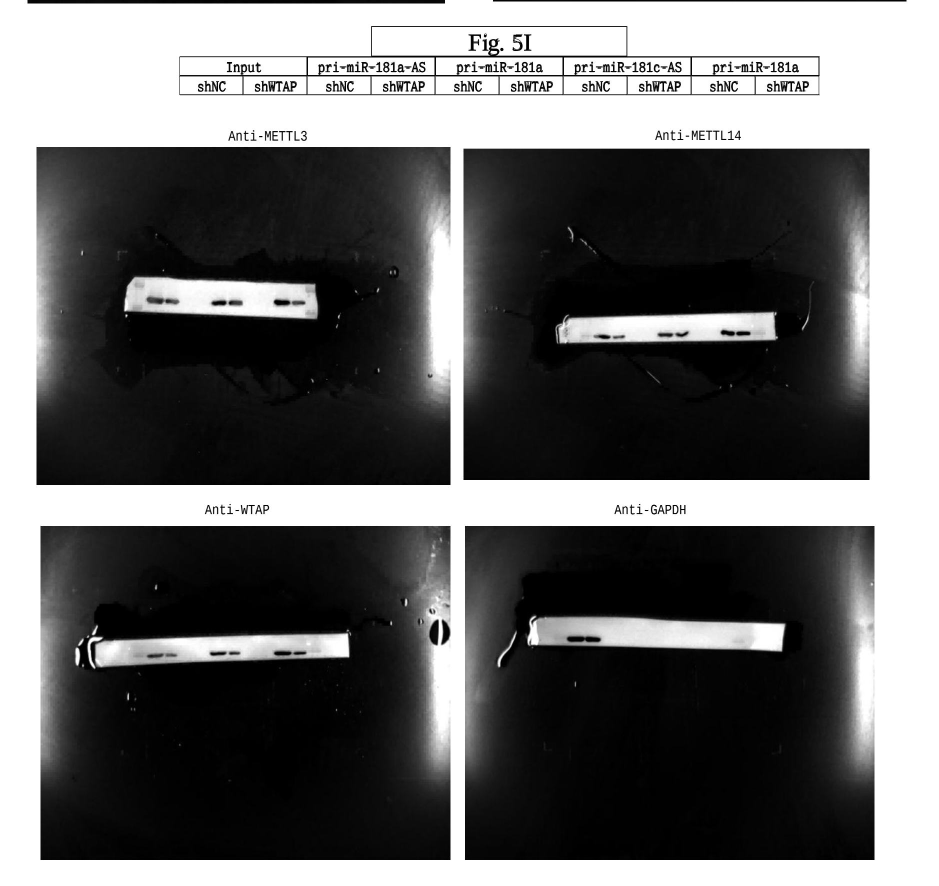

Fig. 6A

Input		pri-miR-181a		pri-miR-181a-m⁶A		pri-miR-181c		pri-miR-181c-m⁶A	
Control	Osteo	Control	Osteo	Control	Osteo	Control	Osteo	Control	Osteo

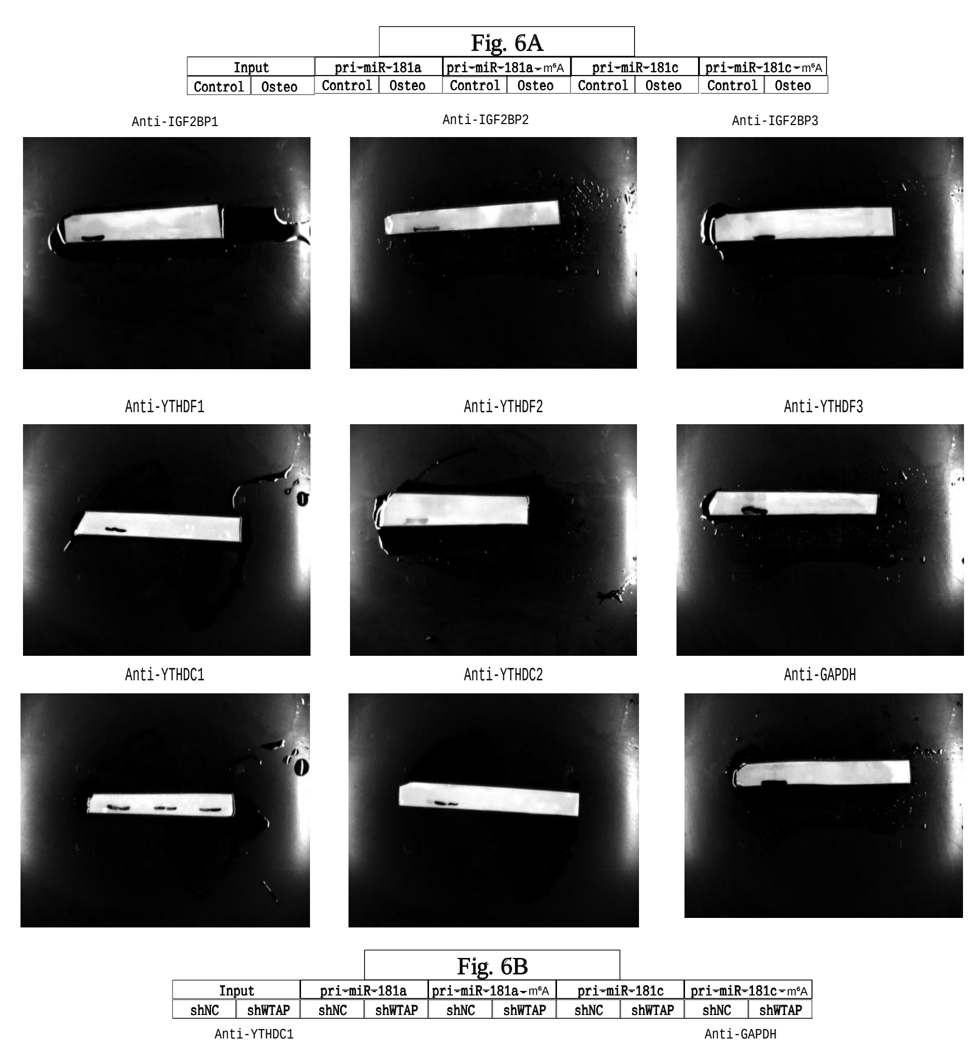

Fig. 6B

Input		pri-miR-181a		pri-miR-181a-m⁶A		pri-miR-181c		pri-miR-181c-m⁶A	
shNC	shWTAP	shNC	shWTAP	shNC	shWTAP	shNC	shWTAP	shNC	shWTAP

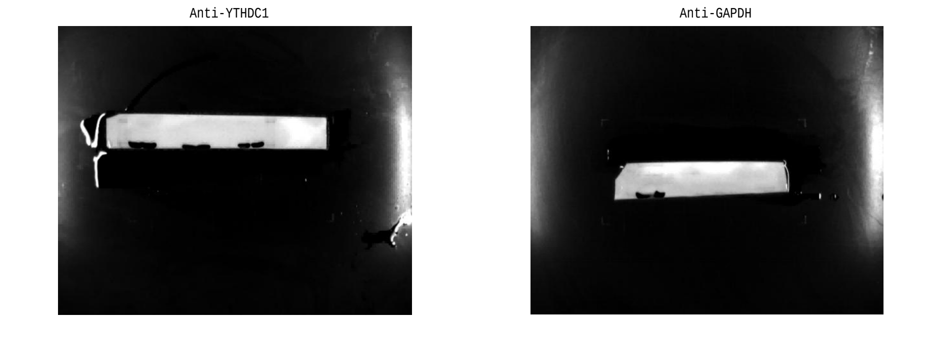

Fig. 6C

Input		pri-miR-181a		pri-miR-181a-m⁶A		pri-miR-181c		pri-miR-181c-m⁶A	
Vector	OE-WTAP	Vector	OE-WTAP	Vector	OE-WTAP	Vector	OE-WTAP	Vector	OE-WTAP

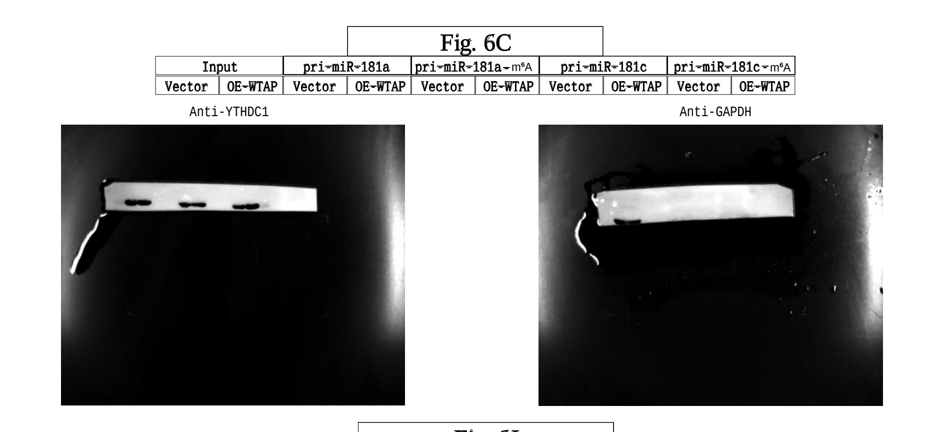

Fig. 6J

siNC	siYTHDC1-1	siYTHDC1-2	siYTHDC1-3

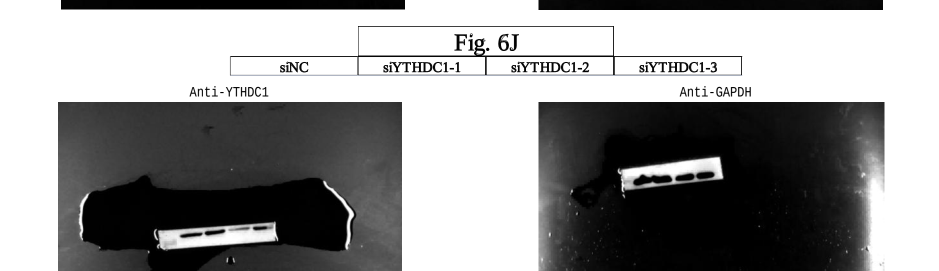

Fig. 6L

siNC siYTHDC1

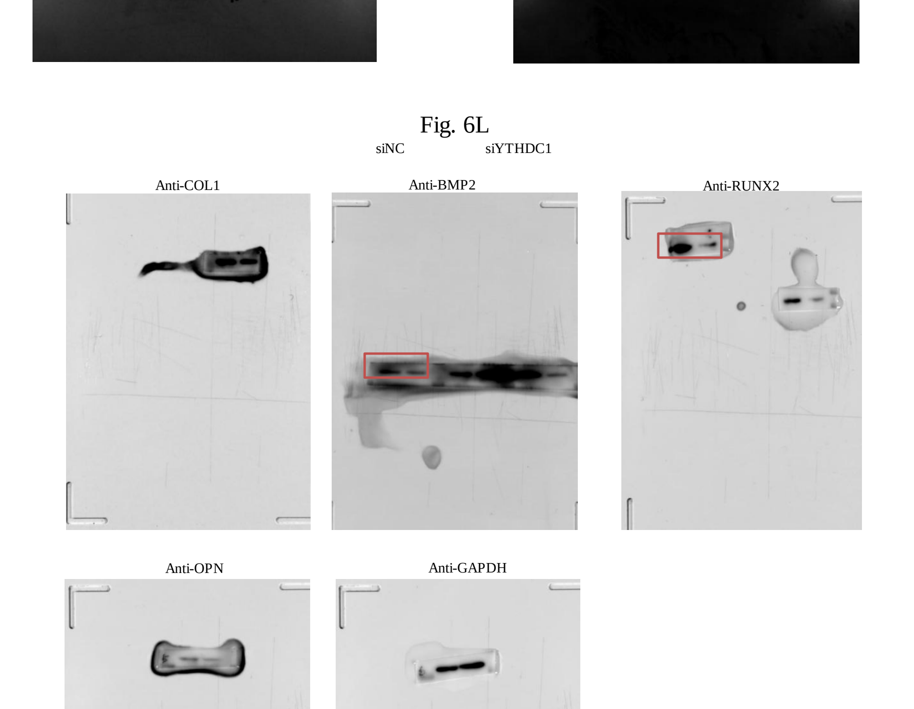

Fig. 6O

siNC siYTHDC1

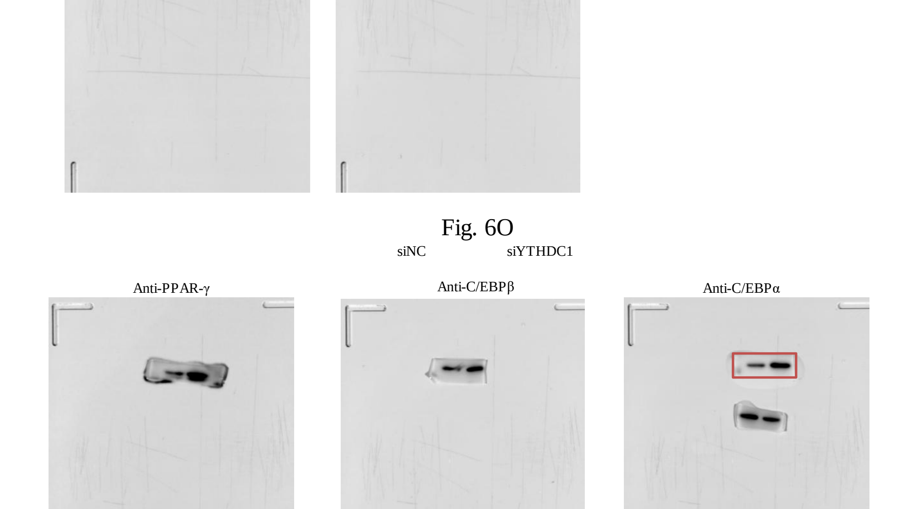

Fig. 7B

Normal Normal Normal Normal OP OP OP OP

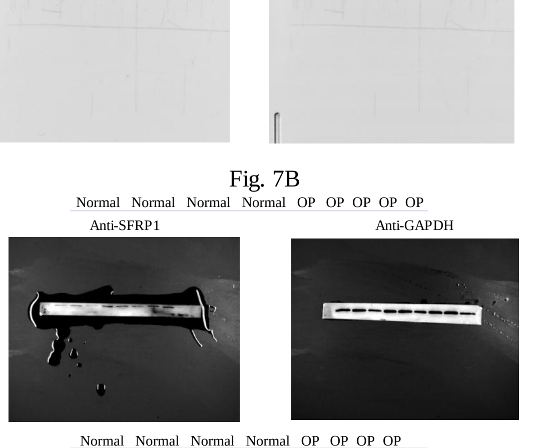

Fig. 7D

Normal Normal OVX OVX

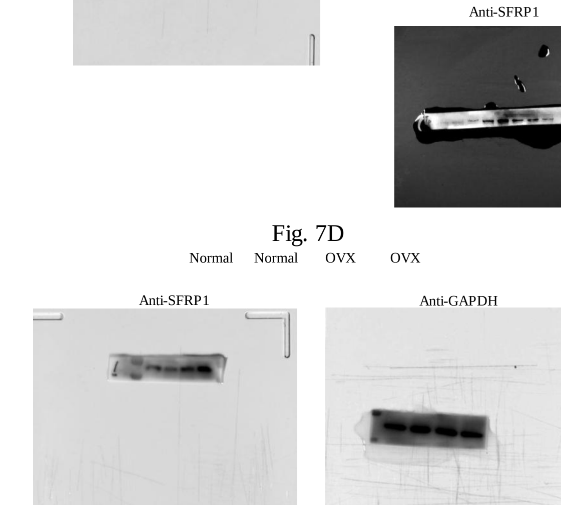

Fig. 7G

Vector OE-WTAP

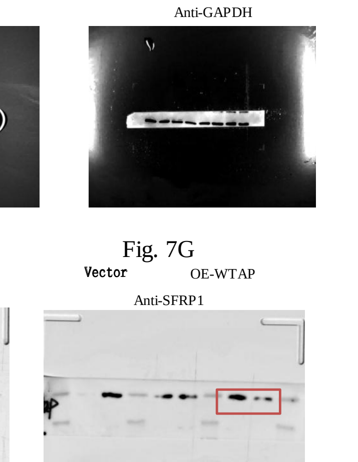

Fig. 7G

Vector OE-WTAP

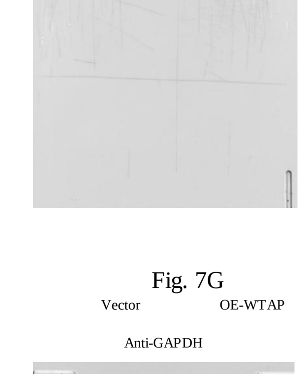

Fig. 7G

shNC shWTAP

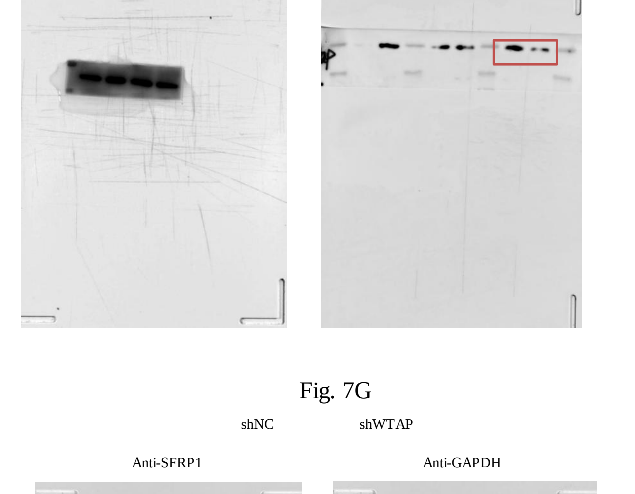

Fig. 7H

NC miR-181a inhibitor NC miR-181a inhibitor

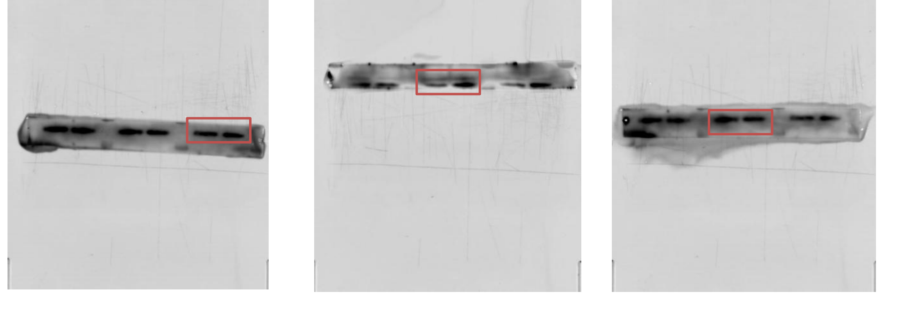

Fig. 7I

NC miR-181c inhibitor NC miR-181c inhibitor

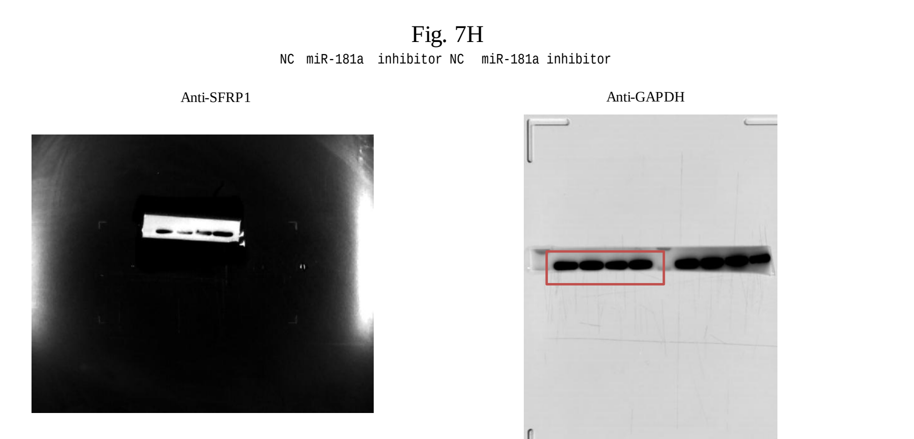

Fig. 7J

Vector	+	-	+	-
OE-WTAP	-	+	-	+
inhibitor NC	+	+	-	-
miR-181a inhibitor	-	-	+	+

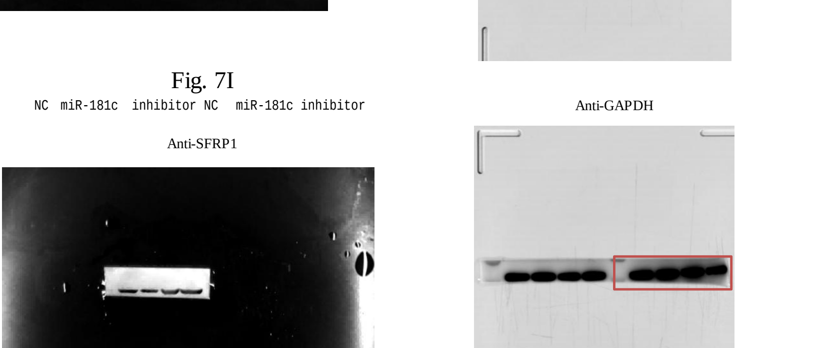

Fig. 7K

Vector	+	-	+	-
OE-WTAP	-	+	-	+
inhibitor NC	+	+	-	-
miR-181c inhibitor	-	-	+	+

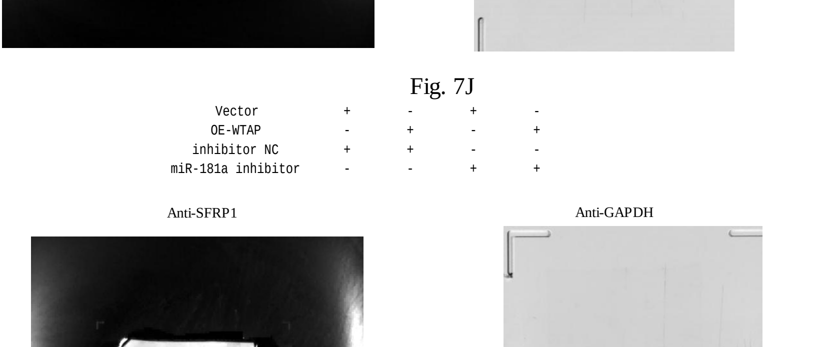

Fig. 7L

shNC	+	-	+	-
shWTAP	-	+	-	+
mimic NC	+	+	-	-
miR-181a	-	-	+	+

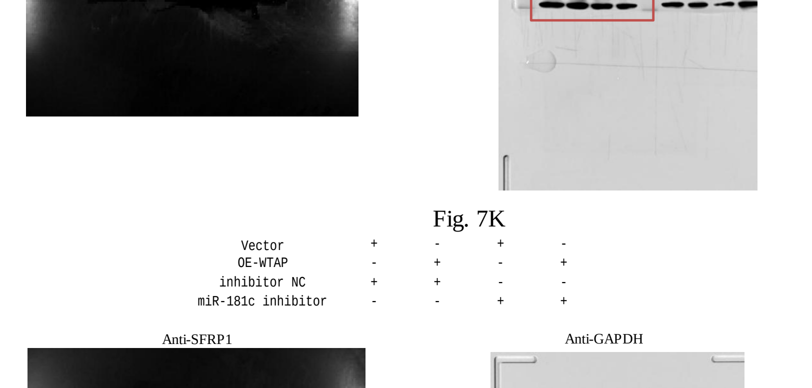

Fig. 7M

shNC	+	-	+	-
shWTAP	-	+	-	+
mimic NC	+	+	-	-
miR-181c	-	-	+	+

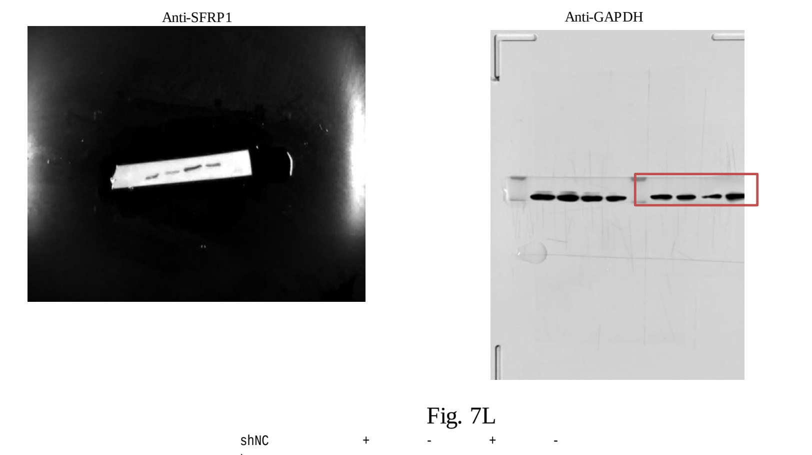

Fig. 7N

siNC siSFRP1-1 siSFRP1-2 siSFRP1-3

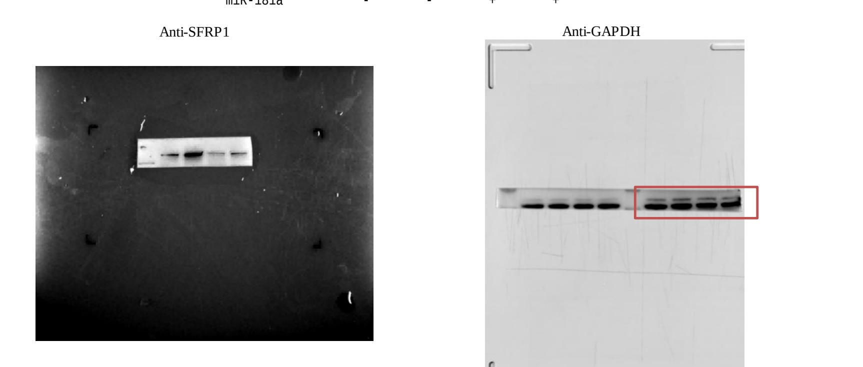

Fig. 7P

siNC siSFRP1

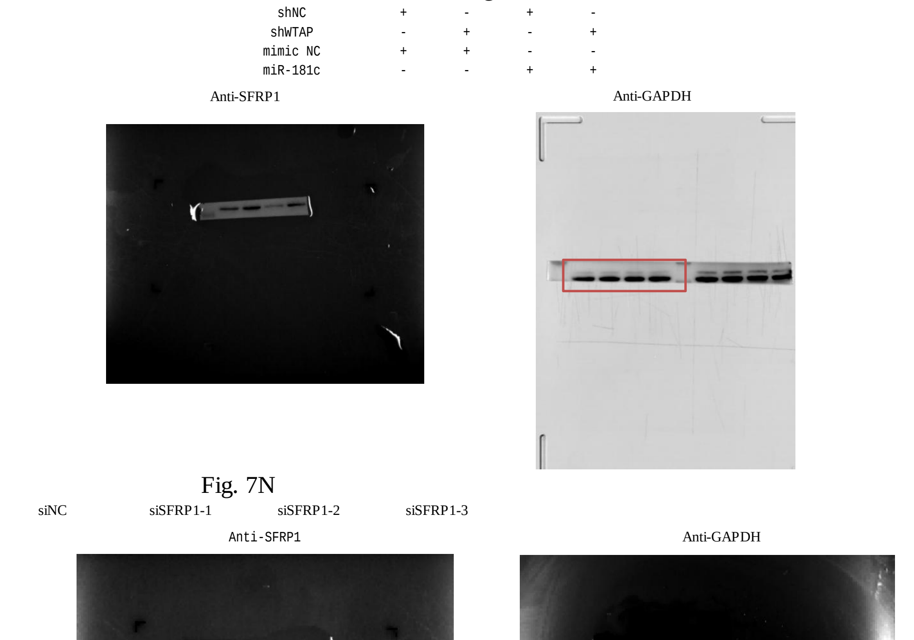

Fig. 7T

siNC siSFRP1

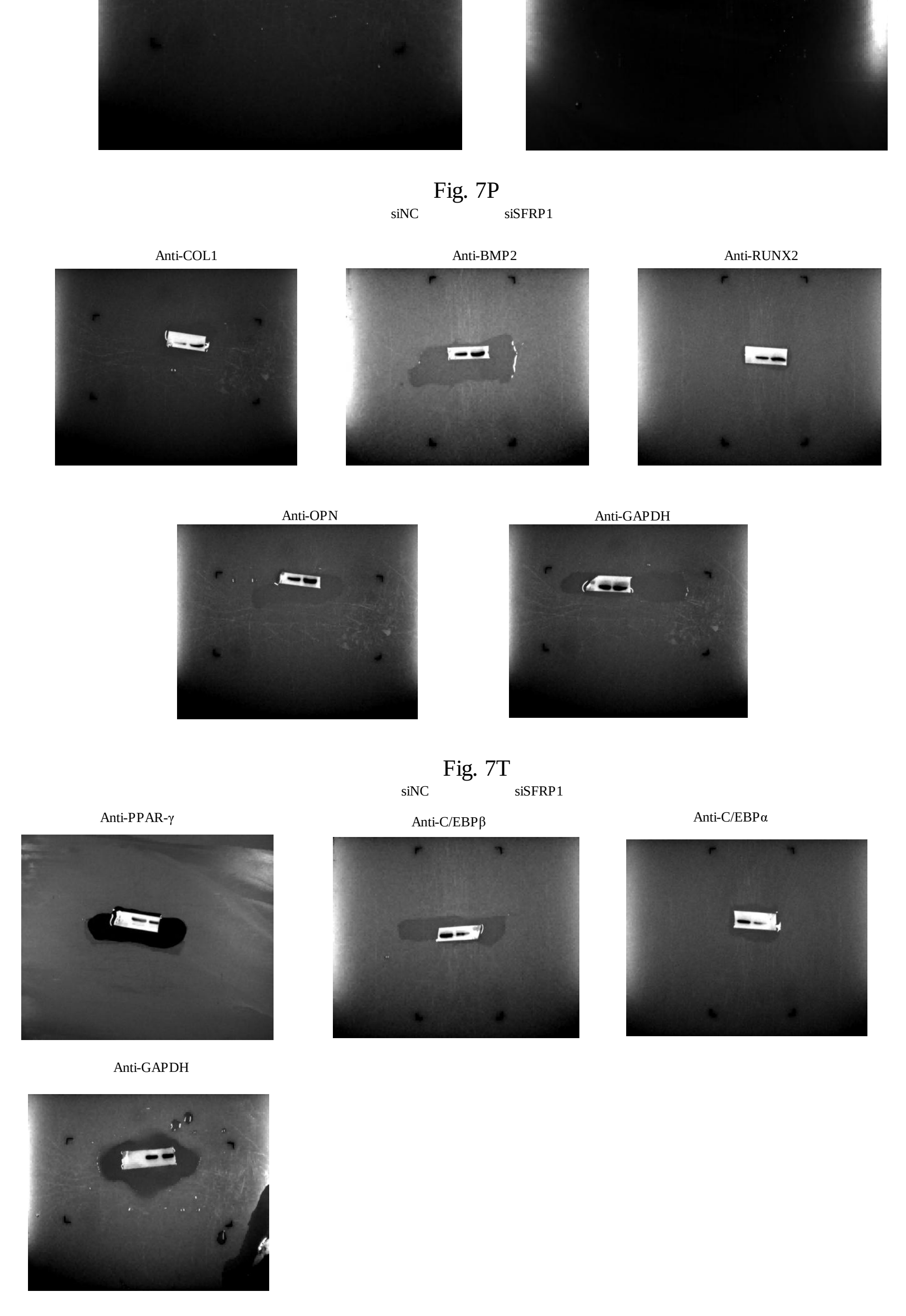

Supplement: Supplementary file 2 — Original Data File [file 41419_2023_5565_MOESM2_ESM.pdf]
